# Tryptophan depletion results in tryptophan-to-phenylalanine substitutants

**DOI:** 10.1038/s41586-022-04499-2

**Published:** 2022-03-09

**Authors:** Abhijeet Pataskar, Julien Champagne, Remco Nagel, Juliana Kenski, Maarja Laos, Justine Michaux, Hui Song Pak, Onno B. Bleijerveld, Kelly Mordente, Jasmine Montenegro Navarro, Naomi Blommaert, Morten M. Nielsen, Domenica Lovecchio, Everett Stone, George Georgiou, Mark C. de Gooijer, Olaf van Tellingen, Maarten Altelaar, Robbie P. Joosten, Anastassis Perrakis, Johanna Olweus, Michal Bassani-Sternberg, Daniel S. Peeper, Reuven Agami

**Affiliations:** 1 Division of Oncogenomics, Oncode institute, The Netherlands Cancer Institute, Amsterdam, The Netherlands; 2 Division of Molecular Oncology and Immunology, Oncode institute, The Netherlands Cancer Institute, Amsterdam, The Netherlands; 3 Department of Cancer Immunology, Institute for Cancer Research, Oslo University Hospital Radiumhospitalet, Oslo, Norway; 4 Institute of Clinical Medicine, University of Oslo, Oslo, Norway; 5 Lausanne Branch, Ludwig Institute for Cancer Research, Lausanne University Hospital (CHUV) and University of Lausanne (UNIL), Lausanne, Switzerland; 6 Center of Experimental Therapeutics, Department of Oncology, Lausanne University Hospital (CHUV), Lausanne, Switzerland; 7 NKI Proteomics facility, The Netherlands Cancer Institute, Amsterdam, The Netherlands; 8 Department of Molecular Biosciences, University of Texas, Austin, TX USA; 9 Division of Pharmacology, The Netherlands Cancer Institute, Amsterdam, The Netherlands; 10 Biomolecular Mass Spectrometry and Proteomics, Bijvoet Center for Biomolecular Research, Utrecht Institute for Pharmaceutical Sciences, Utrecht University and Netherlands Proteomics Centre, Utrecht, The Netherlands; 11 Division of Biochemistry, The Netherlands Cancer Institute, Amsterdam, The Netherlands; 12 Erasmus MC, Department of Genetics, Rotterdam University, Rotterdam, The Netherlands

**Keywords:** Tumour immunology, Proteomics

## Abstract

Activated T cells secrete interferon-γ, which triggers intracellular tryptophan shortage by upregulating the indoleamine 2,3-dioxygenase 1 (IDO1) enzyme^1–4^. Here we show that despite tryptophan depletion, in-frame protein synthesis continues across tryptophan codons. We identified tryptophan-to-phenylalanine codon reassignment (W>F) as the major event facilitating this process, and pinpointed tryptophanyl-tRNA synthetase (WARS1) as its source. We call these W>F peptides ‘substitutants’ to distinguish them from genetically encoded mutants. Using large-scale proteomics analyses, we demonstrate W>F substitutants to be highly abundant in multiple cancer types. W>F substitutants were enriched in tumours relative to matching adjacent normal tissues, and were associated with increased IDO1 expression, oncogenic signalling and the tumour-immune microenvironment. Functionally, W>F substitutants can impair protein activity, but also expand the landscape of antigens presented at the cell surface to activate T cell responses. Thus, substitutants are generated by an alternative decoding mechanism with potential effects on gene function and tumour immunoreactivity.

## Abstract (web summary)

Tryptophan depletion, which occurs in tumours, results in in-frame translation across tryptophan-encoding codons by phenylalanine substitution.

## Main

Activated T cells infiltrating the tumour microenvironment secrete interferon-γ (IFNγ) that induces expression of the enzyme IDO1 in cancer cells. IDO1 catabolizes tryptophan to generate metabolites along the kynurenine pathway to subvert T cell immunity^1–4^. IFNγ also stimulates T cell activity by upregulating human leukocyte antigen (HLA) levels at the cell surface^5^ and increases the repertoire of epitopes by producing aberrant frameshifted proteins through intracellular IDO1-mediated tryptophan depletion^6^. In addition to frameshifting ribosomes^6–8^, amino acid shortages can elicit codon reassignments in bacteria and yeast, thereby altering the decoding of a codon irrespective of its mRNA context to promote in-frame protein synthesis^9–11^. Additionally, codon reassignment due to alternative tRNA aminoacylation has been observed in yeast, where the CUG codon is decoded as both serine and leucine^12^. In mammals, evidence for codon reassignments is so far restricted to methionine codons. Owing to mis-aminoacylation triggered by virus exposure and selenium deficiency, cysteine is incorporated at a UGA stop codon instead of selenocysteine in rat liver cells^13–15^. However, the effects of tryptophan depletion on in-frame protein synthesis are unknown.

## Codon reassignment by tryptophan depletion

**Extended Data Fig. 1:**
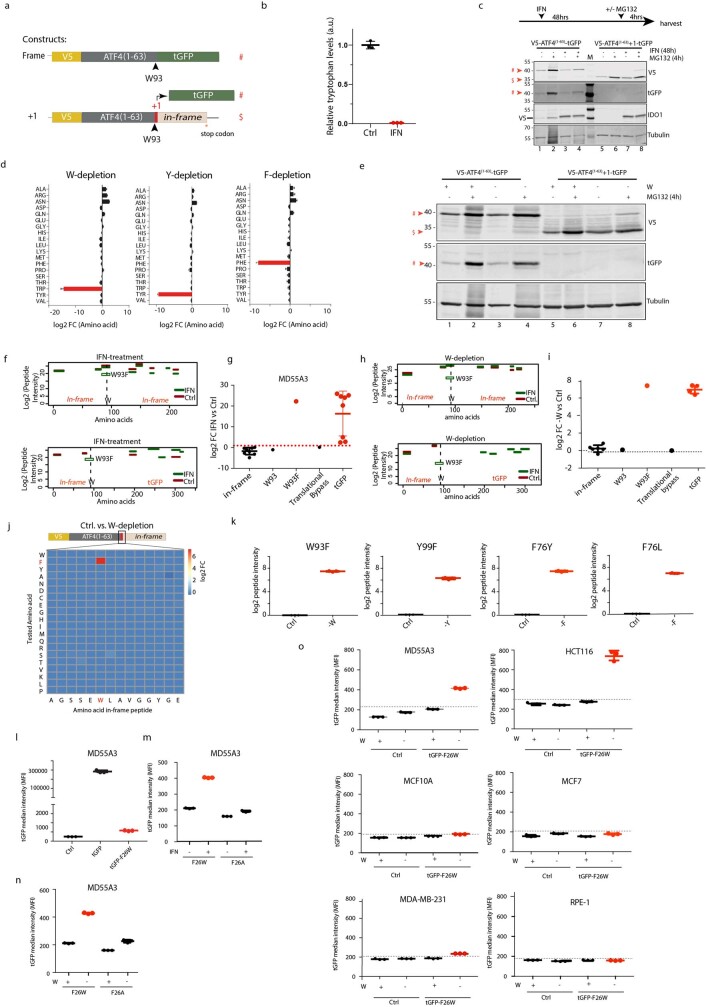
Reporter assays identify IFNγ-induced W>F codon reassignment. (a) A scheme of the reporter V5-ATF4^(1-63)^-tGFP constructs used in this study. The tGFP gene was placed either *in-frame* or +1 nucleotide (nt) *out-of-frame* after the tryptophan codon at position 93 (W93). # and $ mark tGFP-containing and truncated protein products, respectively. (b) Dot plot depicting relative tryptophan levels of MD55A3-V5-ATF4^(1-63)^-tGFP^+1^ cells treated with IFNγ (250U/mL) for 48 h. (c) MD55A3 melanoma cells expressing V5-ATF4^(1-63)^-tGFP and V5-ATF4^(1-63)^-tGFP^+1^ were subjected to IFNγ treatment. Whole cell extracts were subjected to immunoblotting analyses using anti-V5, anti-tGFP, anti-Tubulin and anti-IDO1 antibodies. M: marker lane. Red arrows mark *in-frame* (#) and *out-of-frame* ($) products, as depicted in panel a. (n = 2 independent experiments). (d) Amino acid levels for tryptophan (W), tyrosine (Y), and phenylalanine (F) of the MD55A3-V5-ATF4^(1-63)^-tGFP^+1^ cells depleted of the indicated amino acids, as in Fig. 1d. Each bar represents the average of 3 independent experiments +/- stdev. (e) MD55A3 cells expressing V5-ATF4^(1-63)^-tGFP and V5-ATF4(1-63)-tGFP^+1^ were depleted for tryptophan for 48 h and then treated or not with the proteasome inhibitor MG132 (10μM) as indicated in the scheme in panel b. Whole cell extracts were subjected to immunoblotting analyses using anti-V5, anti-tGFP, and anti-Tubulin antibodies. Red arrows mark *in-frame* (#) and *out-of-frame* ($) products. (n = 2 independent experiments). (f) Line plots depicting the peptides detected following V5-IP/MS of lysates of MD55A3-V5-ATF4^(1-63)^-tGFP^+1^ cells from mock or IFNγ-treated conditions. The dashed line represents the site of the tryptophan 93 codon (W93) and the x-axis represents the *in-frame* part of the reporter protein before and after the tryptophan codon. (g) Dot-plot depicting log2 fold changes between mock and IFNγ-treated MD55A3-V5-ATF4^(1-63)^-tGFP^+1^ cells for the tryptic peptide intensities presented. Each dot represents the intensity of a unique peptide. Lines represent the average intensities of all peptides +/-stdev. (h) The same analysis as performed in panel f, but for mock and tryptophan-depleted MD55A3-V5-ATF4^(1-63)^-tGFP^+1^ cells. (i) The same analysis as performed in panel g, but for mock and tryptophan-depleted MD55A3-V5-ATF4^(1-63)^-tGFP^+1^ cells. (j) Heatmap depicting a log2 fold change between mock and tryptophan-depleted MD55A3-V5-ATF4^(1-63)^-tGFP^+1^ cells for amino acid substitution events for each of the amino acids in the tryptic peptide spanning the W93 codon. (k) Dot-plots depicting peptide intensity values in either mock (Ctrl) or amino acid depleted MD55A3-V5-ATF4^(1-63)^-tGFP^+1^ cells for the specific codon reassignments detected in the various depleted conditions, as indicated. Each dot represents an independent biological replicate and the line represents the average of the 3 replicates +/- standard deviation (stdev). (l-o) tGFP median intensity of reporter vectors encoding for tGFP, tGFP^F26W^, or tGFP^F26A^ stably introduced into the indicated cell lines and conditions. Marked in dotted line is the highest value of the highest point in the background. Each dot represents a biological replicate and the line represents the average of the triplicates +/- stdev.

To examine specific alterations in protein synthesis following tryptophan depletion induced by IFNγ treatment, we used MD55A3 melanoma cells stably expressing V5–ATF4^1^^–63^–tGFP in-frame and +1 out-of-frame reporters^6^. In these vectors, a tryptophan-less turboGFP gene (tGFP) is placed either in-frame or out-of-frame (+1) downstream of an unstable V5-tagged ATF4 fragment comprising residues 1–63 that includes a single tryptophan at position 93 (W93). The +1 vector leads to the expression of a shorter protein owing to a premature stop codon (marked with an asterisk in Extended Data Fig. 1a). We expected that IFNγ-induced tryptophan depletion would block in-frame mRNA translation downstream of the tryptophan codon while inducing the out-of-frame tGFP, as previously reported^6^. However, immunoblotting with anti-V5 revealed the accumulation of both out-of-frame tGFP and in-frame proteins from the +1-construct when intracellular tryptophan was completely depleted after IFNγ treatment (Extended Data Fig. 1b, c). Similar results were obtained in the case of the in-frame construct, and when cells were deprived of tryptophan (Extended Data Fig. 1d, e). The production of tGFP was verified by anti-tGFP staining (Extended Data Fig. 1b, e). The persistent tryptophan-codon-containing in-frame products suggests an alternative decoding mechanism of the ‘hungry codon’.

**Fig. 1:**
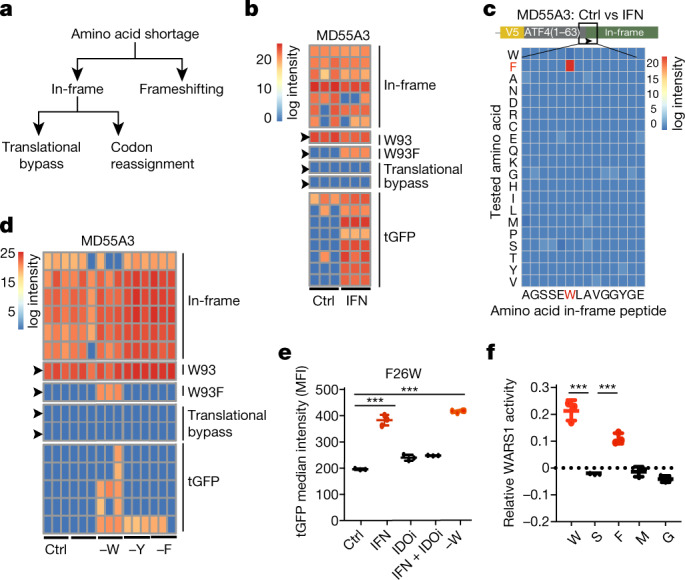
Reporter assays identify IFNγ-induced W>F codon reassignment. **a**, A model depicting possible mechanisms that could allow mRNA translation to proceed in case of amino acid shortages. In addition to ribosomal frameshifting, in-frame translation in the absence of tryptophan could be facilitated by codon reassignment or translational bypass. **b**, MD55A3 melanoma cells expressing V5–ATF4^1–63^–tGFP^+1^ were treated with or without IFNγ (48 h) (IFN) and then immunoprecipitated with anti-V5 and analysed by mass spectrometry. The heat map depicts log_2_ intensities of tryptic in-frame and tGFP peptides, as well as the peptides spanning the W93 codon. Each column represents an independent biological replicate. **c**, Heat map depicting log_2_ differences in intensities between mock- and IFNγ-treated conditions for codon reassignment events for each of the amino acids in the tryptic peptide spanning the W93 codon. This heat map is based on two biological replicates. **d**, Same as **b**, except cells were either mock-treated (Ctrl), or deprived of either tryptophan (−W), tyrosine (−Y) or phenylalanine (−F). **e**, tGFP median intensity of MD55A3 melanoma cells transduced with a vector expressing tGFP(F26W) and subjected to 48 h IFNγ, IDOi and tryptophan depletion (−W) as indicated. Each dot represents an independent biological replicate, the line shows the average and error bars represent ±s.d. ***P < 0.001 by ordinary one-way ANOVA using Bonferroni’s multiple comparison test. **f**, Activity assay of recombinant WARS1 incubated with various amino acids. The dots represent all independent biological replicates, the line depicts the average of the triplicate and bars show s.d. ***P < 0.001 by ordinary one-way ANOVA using Bonferroni’s multiple comparison test.

Possible alternative in-frame decoding mechanisms following amino acid deprivation are codon reassignment and translational bypass. These processes result in the incorporation of a different amino acid than is encoded for in the mRNA and the interruption of translation followed by downstream continuation^9,10,16,17^ (Fig. 1a). Whether these processes occur in human cells is currently unknown. To identify alternative decoding mechanisms experimentally, we performed mass spectrometry analysis of V5-tag-immunoprecipitated proteins (V5-IP/MS) from lysates obtained from either mock- or IFNγ-treated +1 vector-expressing cells. We then searched for control out-of-frame tGFP peptides and for all possible codon reassignment and translational bypass events that may arise at codon W93. We confirmed the presence of tGFP peptides originating from frameshift events following IFNγ treatment, but detected no translational bypass events^6,8^ (Fig. 1b, Extended Data Fig. 1f) . Instead, a specific and exclusive tryptophan to phenylalanine codon reassignment (W93F) appeared only in the IFNγ treatment condition (Fig. 1b, Extended Data Fig. 1f). Globally, although IFNγ treatment decreased the detection of peptides originating from the in-frame sequence, including the peptide spanning the single W93, both out-of-frame tGFP peptides and the W93F peptide were markedly induced (Extended Data Fig. 1g). No other codon reassignment was enriched upon IFNγ treatment at this tryptophan codon or its surroundings (Fig. 1c), indicating this is a major and exclusive translational event. To support the above observations, we depleted the same cells of tryptophan, and performed V5-IP/MS analyses. Here too, we observed specific and exclusive W93F peptides (Fig. 1d, Extended Data Fig. 1h–j). By comparison, depletion of either tyrosine (Y) or phenylalanine (F) did not lead to any W93F, but rather to changes in Y99F (after tyrosine depletion), and F76Y and F76L (after phenylalanine depletion; Extended Data Fig. 1d, k). Together, these results indicate that in the context of our reporter vector, amino acid deprivations induce codon reassignments that are highly specific with respect to the mis-incorporated amino acid.

Next, we sought to address whether tryptophan to phenylalanine codon reassignments (W>F) facilitate the generation of full-length proteins in the absence of tryptophan. For this purpose, we substituted a conserved phenylalanine at position 26 with tryptophan in the otherwise tryptophan-less tGFP (tGFP(F26W)), which abolished the fluorescent signal of this protein (Extended Data Fig. 1l). We, hypothesized that if tryptophan shortage can lead to stable W>F, then green fluorescence should appear following the generation of the mature wild-type protein. Indeed, in response to either IFNγ treatment or tryptophan depletion, the fluorescent signal of tGFP(F26W) increased in comparison to the control (Fig. 1e). The IFNγ-induced tGFP(F26W) signal was negated by IDO1 inhibition (IDOi), pinpointing the causal role of tryptophan shortage (Fig. 1e). In contrast to tGFP(F26W), neither IFNγ treatment nor direct tryptophan depletion of cells containing a control tGFP(F26A) construct affected the green fluorescent signal (Extended Data Fig. 1m, n). Expansion of this analysis to other cell lines indicated a wider phenomenon neither restricted to MD55A3 cells nor to melanoma (Extended Data Fig. 1o). The observation that not all cell lines showed increased tGFP(F26W) signal by tryptophan depletion (that is, MCF7, RPE-1 and MCF10A) may indicate it is a regulated process.

## Role of WARS1 in W>F

The observed induction of W93F, F76Y and Y99F codon reassignments cannot be explained by alternative codon–anticodon interactions. Instead, the aromatic nature of the amino acids involved suggests an error in aminoacylation in the absence of the cognate depleted amino acid. We therefore tested the capacity of recombinant WARS1 to activate amino acids other than tryptophan^11^. WARS1 was able to activate both tryptophan and phenylalanine, but not control amino acids such as serine, methionine and glycine (Fig. 1f). This indicates that phenylalanine is a potential substrate of WARS1 in the absence of tryptophan, and suggests that compromised specificity of amino acid tRNA synthetases may be the source of W>F.

## Proteome-wide W>F

**Fig. 2:**
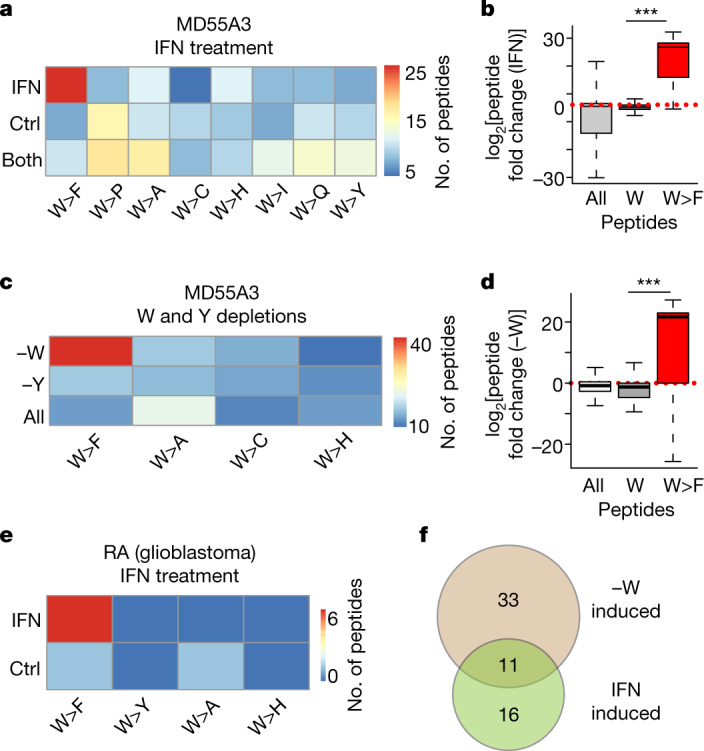
Detection of endogenous W>F substitutants. **a**, Heat map depicting the number of tryptophan (W) codon reassignments detected specifically in mock-treated (Ctrl) MD55A3 V5–ATF4^1–63^–tGFP^+1^-expressing cells, in IFNγ-treated (IFN) cells, or in both. Only the peptides detected in two biological replicates (*n* = 2) of every condition were selected. **b**, Box plot depicting log_2_ fold change in peptide intensities between control and IFNγ-treated condition for all peptides in the proteome (whiskers show range without outliers, boxes encompass first and third quartiles and the centre line indicates the median). The groups are either all peptides detected in the proteome (all), or peptides that span tryptophan codons and contain a tryptophan (W) or W>F, respectively. ***P < 0.001, Wilcoxon test (unpaired two-sample *t*-test). **c**, Same as **a**, but for mock-treated, tryptophan-depleted (−W) or tyrosine-depleted (−Y) MD55A3 V5–ATF4^1–63^–tGFP^+1^-expressing cells (*n* = 2 biological replicates). **d**, Same as **b**, but for log_2_ fold change in peptide intensities between control and tryptophan-depleted conditions. **e**, Heat map depicting the number of the indicated codon reassignment events at tryptophan codons, specifically detected in the proteomes of IFNγ-treated or control glioblastoma RA cells^18^ (*n* = 2 biological replicates). **f**, Venn diagram depicting the overlap between the W>F peptides detected in IFNγ-treated (IFN induced) or tryptophan-depleted (−W induced) MD55A3 cells.

**Extended Data Fig. 2:**
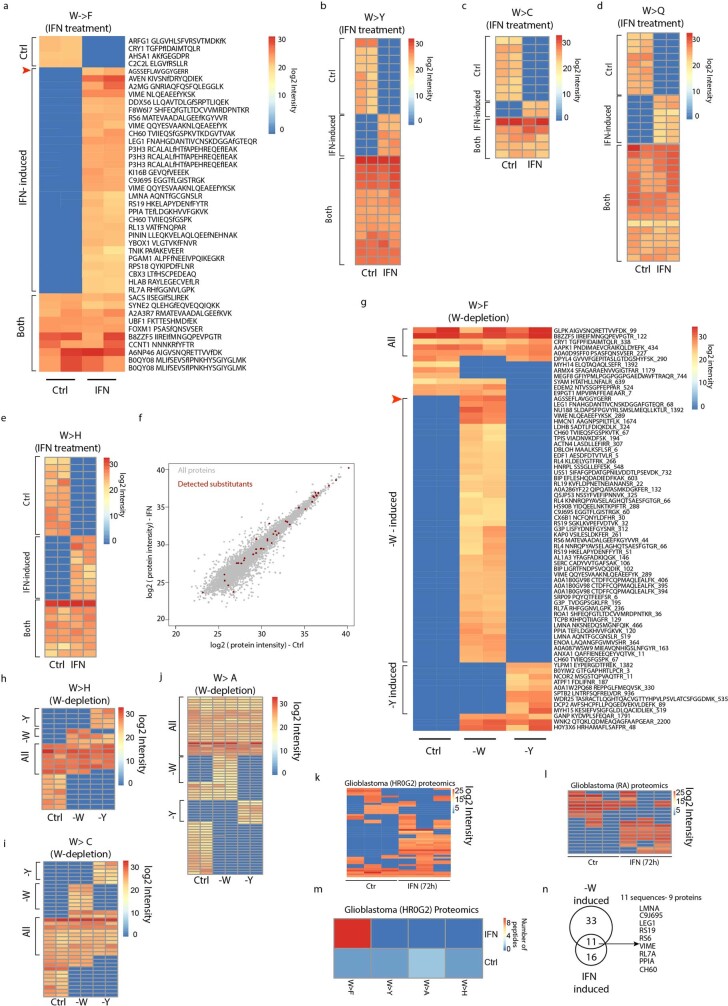
Detection of endogenous W>F substitutants. (a) A heatmap depicting log2 peptide intensities for W>F substitution events in the proteome, selected to be reproducible across two biological replicates (1 column represents 1 replicate) in control (Ctrl) or IFNγ-treated (IFN) MD55A3-V5-ATF4^(1-63)^-tGFP^+1^ cells. Marked with an arrow is the peptide spanning the tryptophan at position 93 (W93) from the V5-ATF4^(1-63)^-tGFP^+1^ reporter. W>F substitutants are marked with a lower case “f”. (b-e) Heatmaps depicting substitutant peptide intensities detected specifically in mock (Ctrl) and IFNγ-treated (IFN) conditions; W>Y (b), W>C (c), W>Q (d) and W>H (e). (f) Scatter plot depicting protein expression calculated as log2 protein intensity from MS analysis in mock-treated (x-axis) and IFNγ-treated (y-axis) MD55A3-V5-ATF4^(1-63)^-tGFP^+1^ cells for all (grey) or substitutant (red) peptides. (g) Same analysis as in panel a, but for tryptophan (-W) and tyrosine (-Y) depletion. (h-j) Same analysis as presented in panels b-e, but for mock (Ctrl), tryptophan depletion (-W), and tyrosine depletion (-Y) conditions, for W>H (h), W>C (i) or W>A (j) comparisons. (k-l) Heatmaps depicting log 2 peptide intensities for W>F substitution events in the proteomes of glioblastoma cell lines HR0G2(k) and RA (l). (m) Heatmap depicting the number of substitutions peptides, as indicated, specifically detected in the proteomes of IFNγ−treated or control glioblastoma HROG02 cells. (n) A Venn diagram (same as Fig. 2f) depicting the overlap between the substitutant peptides specifically detected in IFNγ-treated or tryptophan-depleted MD55A3-V5-ATF4^(1-63)^-tGFP^+1^ cells with the names of the overlapping proteins indicated.

Next, we searched for W>F peptides in endogenous proteins in cells treated with IFNγ. We exposed MD55A3 melanoma cells to IFNγ, performed 2D liquid chromatography with mass spectrometry (LC–MS/MS) in duplicate, and searched for W>F in the entire proteome. After stringent filtering, we identified a significantly higher number of W>F events induced by IFNγ compared with the control (Extended Data Fig. 2a, Supplementary Table 1). This enrichment was unique for W>F, and was not observed for other tested control codon reassignments (Fig. 2a, Extended Data Fig. 2a–e). Additionally, the observed W>F enrichment was significant (*P* < 2.2 × 10^−16^) in the context of reduced global expression of peptides in the whole proteome, probably caused by enhanced proteolysis and reduced mRNA translation by IFNγ (Fig. 2b). Further analysis of the proteins carrying W>F indicated that they are neither highly expressed nor differentially expressed before or after IFNγ treatment (Extended Data Fig. 2f), suggesting that this is a global phenomenon not directly associated with the abundance of proteins.

Similar to IFNγ, tryptophan deprivation caused a specific enrichment of W>F that was not observed for other control codon reassignments (Fig. 2c, Extended Data Fig. 2g, Supplementary Table 2). Furthermore, this W>F enrichment was specifically observed upon tryptophan depletion and not upon tyrosine depletion (Fig. 2c). The enrichment for tryptophan-depletion-induced W>F peptides (44 versus 4) is highly significant, considering the globally reduced protein expression (*P* < 2.2 × 10^−16^; Fig. 2b, d). Specific enrichment of W>F following treatment with IFNγ was also apparent in other cell types^18^ (RA and HROG02 glioblastoma cell lines; Fig. 2e and Extended Data Fig. 2k–m). Together, these data demonstrate a novel mechanism of alternative decoding mechanism following amino acid deprivation in human cells. We therefore named these inducible codon reassignments ‘substitutants’ to distinguish them from genetically encoded mutants.

Notably, 11 W>F substitutants were detected both in tryptophan-depleted and IFNγ-treated MD55A3 cells, confirming their specificity (Fig.2f, Extended Data Fig. 2n). This list included peptides from categories of proteins with different functionalities (for example, RNA binding, ribosome constituents and oxoreductase activity). Of particular interest, the W>F observed in the peptidylprolyl isomerase A (PPIA) protein has already been reported to lose affinity for cyclosporin, leading to its inactivation^19,20^. Also, the W>F in YBX1 protein has been shown to cause decreased binding to C5-methlycytosine (m5C)-containing mRNAs, thereby reducing selectivity towards m5C-containing RNAs^21^. A more global structural analysis of W>F in the detected proteins suggests a wide range of effects enforced by tryptophan shortage on protein activity and function (Supplementary Table 3). Additionally, some of the detected W>F peptides contained two or even three conversions (for example, P3H3; Extended Data Fig. 2a, Supplementary Table 1–3), suggesting effective and iterative W>F at the tryptophan-starved codons.

## W>F substitutants in cancer proteomes

**Fig. 3:**
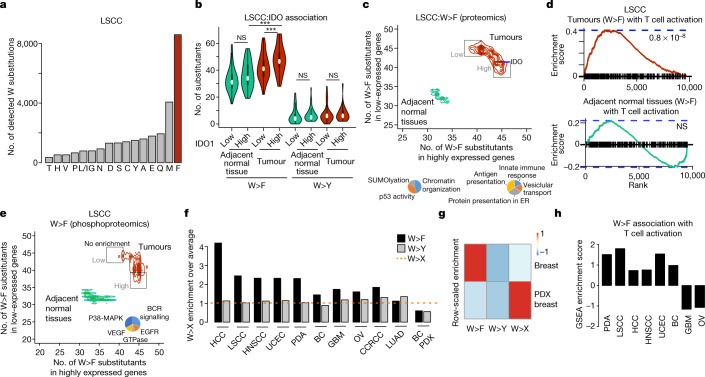
Detection of W>F substitutants in cancer proteomes. **a**, Bar plot depicting cumulative number of tryptophan substitutants detected in the proteomes of LSCC tumour and adjacent normal tissue samples. **b**, Violin plots depicting the number of W>F and W>Y events detected in IDO1 low (intensity < 0) and high (intensity > 0) in LSCC and adjacent normal tissue. Wilcoxon unpaired two-sample *t*-test; ***P = 0.008892 for within-tumour comparison, *P* < 2.2 × 10^−16^ for normal–tumour comparison. **c**, Top, scatter contour plot depicting the number of W>F substitutants per gene when the gene is expressed at a higher (intensity > 0, *x*-axis) or lower (intensity < 0, y-axis) level than in surrounding normal tissue. W>F substitutants in tumours (in red) and normal adjacent normal tissues (in green). Bottom left, pie chart depicting gene ontologies enriched for genes that are expressed at higher level when the number of W>F substitutants is lower in tumour samples. Bottom right, pie chart depicting gene ontologies enriched for genes that are expressed at higher level when the number of W>F substitutants is higher in tumour samples. ER, endoplasmic reticulum. **d**, GSEA plot depicting the enrichment of T cell activation signature stratified against the difference in the number of substitutants in the W>F high class versus the W>F low class. *P*-values by GSEA statistical comparison of ranked distribution against random distribution. **e**, Same as **c**, but for intensities of phosphorylation levels in phosphoproteomics datasets of LSCC and adjacent normal tissues. **f**, Bar plots depicting enrichment of W>F (black) and W>Y (grey) substitutants over the average of all tryptophan substitutants (W>X) in multiple human tumour types. **g**, Row-scaled enrichment heat map for W>F, W>Y and W>X (average) substitutants for primary breast cancer samples xenografts in mouse. **h**, Bar plots depicting GSEA enrichment scores for T cell activation signature stratified against difference in the number of substitutants in W>F high class versus the W>F low class.

**Extended Data Fig. 3:**
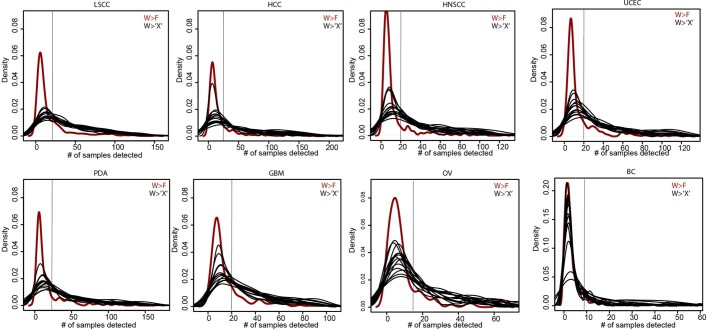
Detection of W>F substitutants in multiple tumour types. Density plots depicting number of samples where the expression of W>F (red) and all other W>’X’ (black) substitutant peptides were detected in multiple tumour types. Vertical lines depict the specificity thresholds used for the selection of substitutants.

To expand our proteomic analyses, we mined the proteomes of the Clinical Proteomic Tumour Analysis Consortium (CPTAC) dataset^22^. Initially, we examined squamous cell lung cancer^23^ (LSCC), a large-scale collection of 205 samples (104 tumours and 101 adjacent normal tissues). Proteomics analysis with highly stringent filtering criteria uncovered a large number of W>F substitutants, which was significantly higher than any other W>X possibilities (Fig. 3a). The detected W>F were expressed less consistently across samples as compared to any other substitutants (Extended Data Fig. 3), suggesting variability in their expression across samples.

Since the LSCC proteomics dataset contained tumours as well as adjacent normal tissues, we next examined the relative enrichment of W>F substitutants and stratified the samples by IDO1 expression. This analysis identified a significantly higher number of W>F events in tumours compared with adjacent normal tissues (Fig. 3b). Moreover, the association of W>F substitutants to IDO1 expression level was specific to tumours and not seen in normal tissues (Fig. 3b). In contrast, W>Y substitutions showed neither enrichment in number, nor association to tumours or IDO1 expression (Fig. 3b).

**Extended Data Fig. 4:**
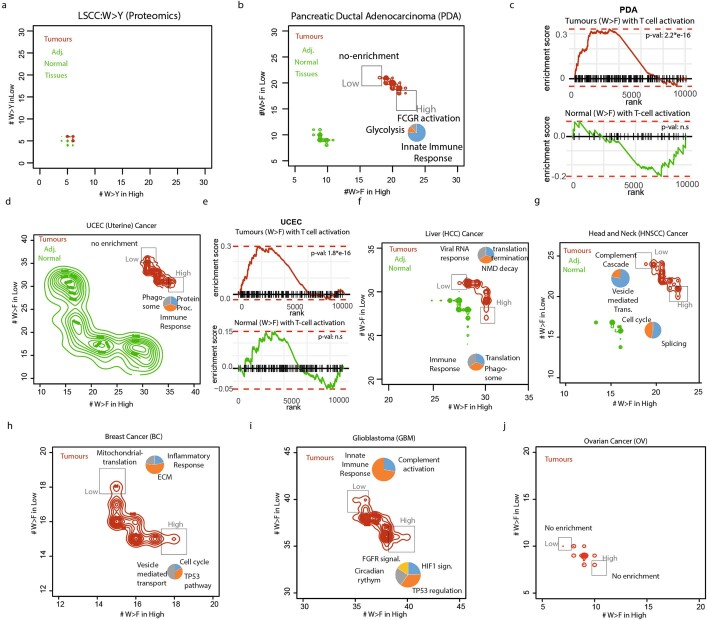
Detection of W>F substitutants in cancer proteomes. (a) Analysis of LSCC tumour proteomes: A scatter contour plot depicting for every gene the number of W>Y peptides when the gene is higher expressed (intensity > 0) on X-axis (High Class) and when the gene is lower expressed (intensity < 0) on Y-axis (Low Class). Contours depict the density of the distribution. W>Y peptides in tumours and normal adjacent normal tissues are depicted in red and green, respectively. Inset: (HIGH) Pie chart depicting representative gene ontologies for genes that are higher expressed when the number of W>Y peptides is high in tumour samples. (LOW) Same as HIGH, but enriched for genes that are higher expressed with W>Y peptides is low. (b) Same as in panel a but for PDA tumour proteomes and for W>F peptides. (c) Gene Set Enrichment Analysis (GSEA) plot depicting the enrichment of T cell activation signature stratified against the difference in the number of substitutants in W>F High Class versus the W>F Low Class for PDA tumour and tumour-adjacent normal tissue dataset. P-values are calculated by GSEA statistical comparison of ranked distribution against random distribution. (d) Same as panel b but for UCEC tumour proteomes. (e) Same as panel c but for UCEC tumour proteomes. (f) Same as panel b but for Liver (HCC) Cancer. (g) Same as panel b but for Head and Neck (HNSCC) Cancer. (h) Same as panel a but for Breast Cancer (BC) tumours only. (i) Same as panel a but for Glioblastoma (GBM) tumours only. (j) Same as panel a but for Ovarian (OV) cancer.

Spurred by this result, we searched for gene-expression signatures associated with the occurrence of W>F substitutants in tumours relative to adjacent normal tissues. We calculated the number of substitutants in tumour samples with high (log intensity greater than 0) and low (log intensity less than 0) expression for every gene. This analysis led to clustering of genes into three categories (Fig. 3c, red). The major cluster represents the vast majority of genes that do not associate with the W>F substitutants landscape. The other two clusters represent genes whose expression is either relatively high in tumours with a high number of W>F substitutants (W>F high), or relatively low in tumours with a high number of W>F substitutants (W>F low). Examination of gene ontologies enriched in the W>F high cluster linked this type of substitutants with a high immune response, vesicular transport and peptide presentation (Fig. 3c, Supplementary Table 4, 5). By contrast, ontologies related to chromatin, p53 activity and SUMOlyation were enriched in the W>F low cluster (Fig. 3c). Furthermore, adjacent normal tissues showed no clustering or enrichment with any gene-expression signature, indicating that W>F substitutants and their associated pathways are a tumour-specific phenomenon (Fig. 3c, green). Finally, a similar analysis of control W>Y substitutions showed no association with gene-expression clusters, providing evidence for the specific appearance of W>F substitutants in human tumours (Extended Data Fig. 4a). Gene set enrichment analysis (GSEA)^24^ further substantiated the link between the occurrence of W>F substitutants and T cell activation index, specifically in tumours and not in adjacent normal tissues (Fig.3d). Analysis of LSCC phosphoproteomic datasets (Supplementary Table 6) linked the expression of W>F substitutants in tumours but not in adjacent normal tissues to p38 MAPK, BCR, EGFR and VEGF kinase signalling pathways (Fig. 3e).

An independent proteomics analysis of pancreatic ductal adenocarcinoma^25^ (PDA) supported a more specific expression of W>F compared with other substitutions (Extended Data Fig. 3), and linked the expression of W>F substitutants in tumours with high levels of infiltrating T cells (Extended Data Fig. 4b, c, Supplementary Tables 5, 7).

Observing W>F substitutants in two independent tumour types led us to complete the analysis of all available tumour types present in CPTAC database, including liver^26^ (HCC, 331 samples), head and neck^27^ (HNSCC, 158 samples), uterine carcinoma^28^ (UCEC, 149 samples), breast cancer^29^ (BC, 108 samples), glioblastoma^30^ (GBM, 111 samples), ovarian cancer^31,32^ (OV, 107 samples), renal cancer^33^ (CCRCC, 194 samples) and lung adenocarcinoma^34^ (LUAD, 217 samples) . Figure 3f shows that W>F is highly enriched (in comparison to average W>X substitutants) in multiple tumour types, and is significantly higher than W>Y in every case except for LUAD. Thresholding for the fold differences between enrichment of W>F and W>Y events classified all tumour types except CCRCC and LUAD as enriched in W>F substitutants (Fig. 3f, Extended Data Fig. 3), indicating that it is a widespread phenomenon in human cancer.

To support the link between tumour immunological microenvironment and W>F expression we analysed a dataset of patient-derived breast cancer xenografts (PDX) expanded in immunodeficient mice^35^ (27 samples). Unlike breast cancers in their native microenvironment, PDX samples did not show an enrichment of W>F substitutants (Fig. 3g), indicating that the host tumour microenvironment and a compatible immune response are required for W>F substitutants. This observation is strengthened by the link between the T cell activation pathway and the occurrence of W>F substitutants in all tumour types except GBM and OV (Fig. 3h, Extended Data Fig. 4d–j, Supplementary Table 6).

Together, global analysis of multiple panels of cancer proteomes and phospho-proteomes exposed the specific appearance of W>F substitutants and uncovered molecular pathways associated with their efficient expression. Intra-tumour T cell activity, high level of IDO1, and certain oncogenic signalling pathways are thus proposed to stimulate W>F substitutants.

## Substitutants lead to immune recognition

**Fig. 4:**
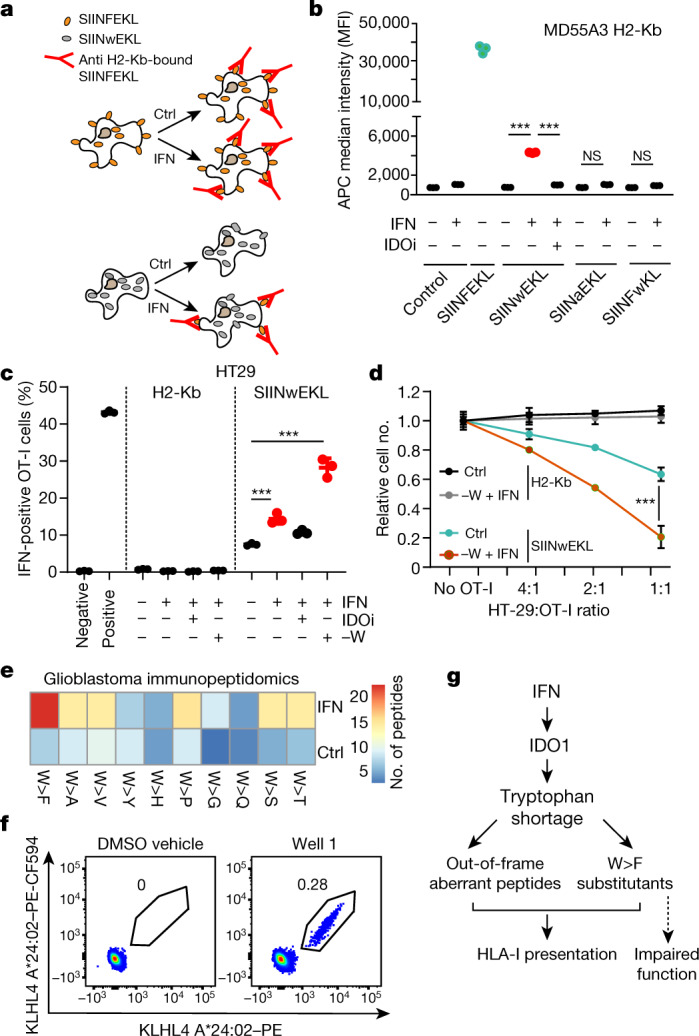
Substitutants are presented at the cell surface and activate T cells. **a**, A model predicting the effect of IFNγ on the presentation and recognition of SIINFEKL and SIINwEKL by anti-H2-Kb-bound SIINFEKL antibodies. **b**, Dot plot depicting the APC median fluorescence intensity (MFI) of H2-Kb-bound SIINFEKL peptides in MD55A3 cells expressing H2-Kb (MD55A3 H2-Kb) in combination with the V5–ATF4^1–63^–tGFP–SIINxxKL reporters. Each dot represents an independent biological replicate (*n* = 3). ***P < 0.001, ordinary one-way ANOVA using Sidak’s multiple comparison test. **c**, HT29 H2-Kb control and SIINwEKL-expressing cells were pre-treated with IFNγ, IDOi and tryptophan depletion as indicated and used in co-cultures with OT-I-derived T cells for 4 h. T cell activation was assessed by flow cytometry analysis of intracellular IFNγ positivity. Dots represent values obtained from independent experiments. Lines represent mean ± s.d. of three independent experiments. ***P < 0.001, ordinary one-way ANOVA using Sidak’s multiple comparison test. **d**, Tumour-killing efficacy of OT-I T cells in co-cultures with control HT29 H2-Kb or HT29-H2-Kb-SIINwEKL cells. Dots represent the mean relative cell number of the indicated co-cultures plus s.d. of three independent experiments. ***P < 0.001 by ordinary one-way ANOVA using Sidak’s multiple comparison test. **e**, Immunopeptidomics analysis of mock and IFNγ-treated RA glioblastoma cells. Heat map depicting the number of various substitutions detected in immunopeptidomics analysis of mock (Ctrl) or IFNγ-treated RA glioblastoma cells. Only the peptides identified in two biological replicates were counted. **f**, Flow cytometric analysis of CD8^+^ T cells following co-culture of naive CD8^+^ T cells and autologous monocyte-derived dendritic cells pulsed with peptide or DMSO vehicle. Plots show T cells reactive to streptavidin–PE and streptavidin–PE-CF594-labelled pMHC multimers complexed with the KLHL4 substitutant peptide YFDPHTNKF (well 1). **g**, A model depicting the induction of W>F substitutants following tryptophan depletion associated to IFNγ treatment and T cell activation.

**Extended Data Fig. 5:**
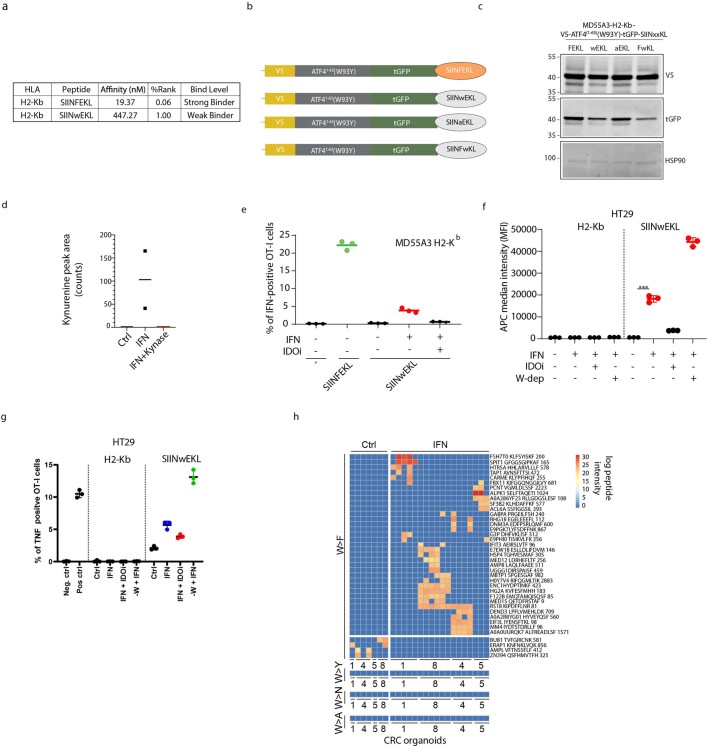
Presentation of substitutants at the cell surface. (a) The binding affinity of SIINFEKL and SIINwKEL peptides to H2-Kb receptor was assessed by NetMHC 4.0. (b) A scheme of the reporter vectors used to assess the production, presentation and T cell activation by SIINFEKL and SIINwEKL. (c) Immunoblot analyses using anti-V5, anti-tGFP and anti-HSP90 antibodies demonstrate similar expression of the various tGFP-SIINxxKL reporters used in this study. Results are representative of 2 independent experiments. (d) MD55A3-V5-ATF4^(1-63)^-tGFP^+1^ cells were either treated with mock (Ctrl), IFNγ (IFN), or IFNγ with recombinant kynureninase (IFN+Kynase) for 48 h. Kynurenine levels in the medium were analysed by MS. Dots represent measurements relative to control from independent experiments, with the line indicating the mean of all samples. (e) MD55A3-H2-Kb control, SIINFEKL or SIINwEKL expressing cells, were pre-treated for 48 h with IFNγ (250U/mL) and IDOi (300 μM) as indicated, and used in co-cultures with OT-I-derived T cells for 12 h. T cell activation was assessed by flow cytometry analysis of intracellular IFNγ positivity. Dots represent values obtained from independent experiments. The lines represent the average of three independent experiments +/- stdev. *** P<0.001 by ordinary one-way ANOVA using Sidak’s multiple comparison test. (f) A dot plot depicting the APC median fluorescence intensity (MFI) of H2-Kb-bound SIINFEKL peptides in HT29 cells expressing H2-Kb (HT29-H2-K^b^) or in combination with the V5-ATF4^(1-63)^-tGFP-SIINwEKL reporter. Each dot represents an independent biological replicate (n = 3). *** P<0.001 by ordinary one-way ANOVA using Sidak’s multiple comparison test. (g) Dot plot representing the percentage of TNFα positive OT-I T cells after 4 h of co-culture with HT29-H2-Kb or H2-Kb-SIINwEKL expressing cells pre-treated 48 h IFNγ (IFN, 250U/mL), with or without IDOi (300 μM) and W-depletion, as determined by flow cytometry. Dots represent values obtained from independent experiments. The lines represent the average of three independent experiments +/- stdev. (h) A heatmap depicting log2 peptide intensities in either control or IFN-treated patient-derived colorectal cancer organoid models for W>F, W>Y, W>N and W>A substitutant peptides.

Next, we tested whether the induction of W>F substitutants following IFNγ treatment can alter the landscape of antigens presented at the cell surface. We used the model peptide SIINFEKL from chicken ovalbumin, which binds mouse H2-Kb and can be detected in this context by a monoclonal H2-Kb-bound SIINFEKL antibody in a flow cytometry assay^36^. The SIINFEKL peptide contains a phenylalanine residue at its centre, and can therefore be exploited to examine the effect of W>F substitutants on the presentation of peptides at the cell surface (Fig. 4a). The H2-Kb binding affinity prediction using NetMHC4.0 server^37^ indicated that although SIINFEKL is a strong binder to H2-Kb (affinity < 20 nM), SIINwEKL is a weak binder (affinity < 400 nM) (Extended Data Fig. 5a). We therefore engineered MD55A3 melanoma cells to express H2-Kb, and SIINFEKL, SIINwEKL, SIINaEKL or SIINFwKL downstream of V5–ATF4^1–63^(W93Y)–tGFP, (Extended Data Fig. 5b). Immunoblot analysis with V5 or anti-tGFP antibodies reveals similar intracellular levels of all protein products (Extended Data Fig. 5c). Flow cytometry analysis indicated that whereas SIINFEKL resulted in the expected antibody recognition at the cell surface of untreated cells, the SIINwEKL peptide did not (Fig. 4b). As predicted from W>F substitutants by tryptophan depletion, a significant SIINFEKL signal was detected in the IFNγ-treated SIINwEKL cells, which was negated by IDOi treatment (Fig. 4b). By contrast, control alternative genetically encoded SIINFEKL mutations showed no signal.

To examine the ability of T cells to recognize the presentation of IFNγ-induced substitutants, we co-cultured MD55A3-H2-Kb-SIINFEKL and SIINwEKL cells with T cells derived from OT-1 mice (harbouring the anti-SIINFEKL transgenic T cell receptor^36^), and examined their reactivity by measuring intracellular IFNγ accumulation. To prevent T cell inactivation by IFNγ-induced kynurenine, we supplemented samples with kynureninase^38^ (Extended Data Fig. 5d). SIINFEKL-presenting cells induced a response in about 20% of the T cells, whereas SIINwEKL-producing cells did not induce any response. Of note, treatment of MD55A3-H2-Kb-SIINwEKL cells with IFNγ led to T cell activation of approximately 5% of the T cells (Extended Data Fig. 5e). This effect was negated by the addition of IDOi, indicating dependency on IFNγ-induced tryptophan depletion by IDO1. Similar but more potent results were obtained with the HT29 colorectal cancer cell line, where a combined treatment of IFNγ with tryptophan depletion showed an especially strong W>F substitution presentation and T cell recognition (Fig. 4c, Extended Data Fig. 5f, g). Notably, whereas SIINwEKL presentation induced only weak T cell recognition (Fig. 4c, Extended Data Fig. 5e), the extent of SIINwEKL>SIINFEKL substitutants was sufficient to stimulate very efficient T cell killing of cells that were pre-treated with IFNγ and tryptophan depletion (Fig. 4d). Thus, W>F substitutants produced by tryptophan depletion can result in antigen presentation at the cell surface that can lead to potent T cell recognition, activation and killing of cancer cells.

## W>F substitutants by immunopeptidomics

Following these SIINwEKL experiments, we next sought to examine the cell surface presentation of endogenous peptides with W>F substitutants. First, we searched for these peptides in a published immunopeptidomics dataset of four microsatellite stable colorectal cancer organoids^39^ treated or not treated with IFNγ for 48 h. IFNγ treatment did not expand the neoantigen landscape of these organoids^39^; however, we observed IFNγ-treatment-specific induction of W>F substitutants (Extended Data Fig. 5h). After filtering (Supplementary Table 8), we observed 41 W>F substitutants across organoids upon IFNγ treatment, as opposed to only 4 in control conditions (Extended Data Fig. 5h). By contrast, a search for W>Y, W>N and W>A, substitutions revealed no detectable peptides (Extended Data Fig. 5h). The variability in the identified W>F substitutant peptides across organoids can be explained by the diversity of HLA class I molecules expressed in each of them. Of interest, organoids number 4 and 8 have several HLA molecules in common (HLA-A*03:01, HLA-C*04:01 and HLA-C*05:01) and share one W>F substitutant peptide originating from the RPS18 gene (Extended Data Fig. 5h, KIPDfFLNR, where f marks the W>F event). This substitutant reproducibly appeared in all ten biological replicates of these two organoids. Even though the samples were from different tissues (colorectal versus melanoma) and the experiments were set up differently (proteomics versus peptidomics), the same W>F substitutant was observed in the proteomics analysis of melanoma MD55A3 cells after IFNγ treatment (QYKIPDfFLNR; Fig. 2a, Extended Data Fig. 2a).

**Extended Data Fig. 6:**
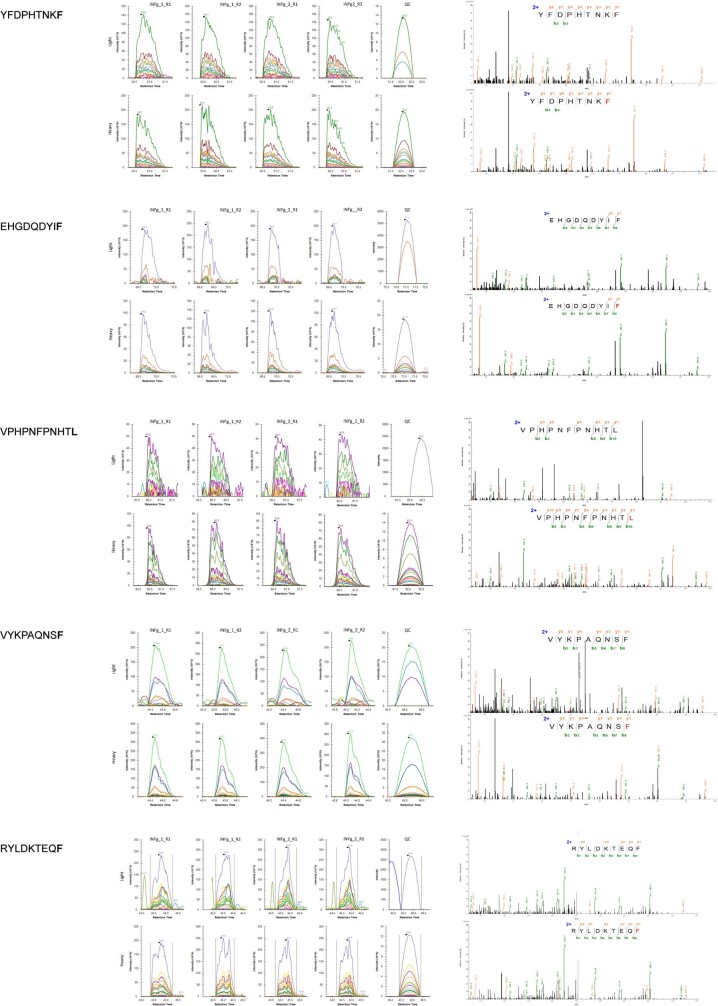
Validation of five substitutants epitopes identified in RA cells. The identification of five substitutant peptides was validated with targeted mass spectrometry. The co-elution profiles of the transitions of spiked-in synthetic heavy-labelled peptides and endogenous eluted HLA bound peptides are shown in two biological replicates of RA cells treated with IFNγ (each in two technical replicates R1 and R2). The quality control (QC) analysis of the pool of synthetic peptides is provide for comparison. The MS/MS fragmentation pattern further confirms the presence of the endogenous peptide (Δm = 10 Da for F and Δm = 7 Da for L). Labelled amino acids are shown in bold.

**Extended Data Fig. 7:**
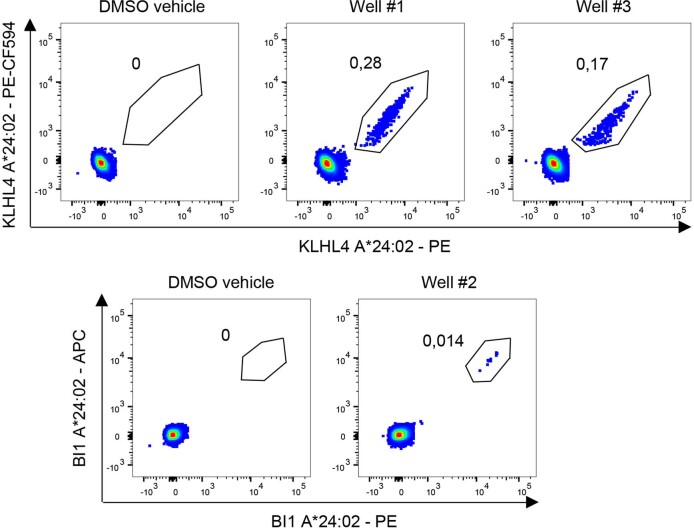
T cell reactivity against two substitutant peptides. Flow cytometric analysis of CD8+ T cells following co-culture of naive CD8^+^ T cells and autologous monocyte-derived dendritic cells pulsed with peptide or DMSO vehicle. Plots show T cells reactive to SA-phycoerythrin (PE) and SA-phycoerythrin-CF594 (PE-CF594)-labelled pMHC multimers complexed with the KLH4L substitutant peptide YFDPHTNKF (Wells #1 and 3), or reactive to SA-PE and SA-allophycocyanin (SA-APC)-labelled pMHC multimers complexed with the BI1 (TMBIM6) substitutant peptide EHGDQDYIF (Well #2).

Second, we searched for the appearance of W>F substitutants in the immunopeptidome of the glioblastoma cell line RA, in which we previously detected their enrichment in the proteome following IFNγ treatment (Fig. 2f, Extended Data Fig. 2m, Supplementary Table 9). Indeed, specific enrichment of W>F, but not control substitutions, appeared in the immunopeptidome of RA cells following IFNγ treatment (Fig. 4e). The identification of five substitutant epitopes was validated with targeted mass spectrometry analysis where synthetic standard peptides labelled with stable heavy isotopes were spiked into newly generated samples of HLA-bound peptides eluted from RA cells treated with IFNγ (Extended Data Fig. 6). We then tested whether these substitutant epitopes can be immunogenic. On the basis of HLA restriction (RA expresses HLA-A*24:02), we selected 6 substitutant epitopes from the 21 identified peptides and tested whether they can prime naive CD8^+^ T cells isolated from healthy HLA-A*24:02^+^ donors^40^. Monocyte-derived dendritic cells isolated from peripheral blood mononuclear cells (PBMCs) from healthy individuals were pulsed with substitutant peptides and co-cultured with autologous naive CD8^+^ T cells. After co-culture, combinatorial peptide–MHC multimer staining followed by flow cytometry analyses showed T cell reactivity towards the two substitutant KLHL4- and TMBIM6-derived peptides that have been confirmed by the targeted mass spectrometry analysis (Fig. 4f and Extended Data Fig. 6, 7). Even though reactivity against wild-type peptides was not tested, these results demonstrate the potential immunogenicity of substitutants.

In sum, we describe here the presence of substitutants—proteins with site specific codon reassignments that appear following amino acid deprivation (Fig. 4g). Whereas genetic mutations give rise to fixed changes in proteins, substitutants are inducible protein alterations that arise owing to errors at the mRNA translational level. We show that W>F substitutants are produced by tryptophan shortage induced by IFNγ-mediated IDO1 induction, and can be processed and presented by HLA class I at the cell surface, and activate T cells. The appearance of W>F substitutants is enriched in tumours compared with adjacent normal tissues, and is associated with increased immune reactivity and oncogenic signalling. Moreover, IFNγ-induced W>F substitutants add a new layer to the landscape of the immunopeptidome presented on cancer cells. In particular, in contrast to the scarcity of epitopes with amino acid substitutions arising from genetic mutations, a substantial enrichment of W>F substitutants was observed in the immunopeptidome of IFNγ-treated microsatellite stable colorectal cancer organoids. The observation that W>F substitutants are shared across tumours can potentially lead to generic therapeutic strategies as compared with the largely private genetically encoded neoantigenic repertoire. Beyond cancer immunology, we also show that substitutant proteins are constituents of the stable proteome, and indicate that they may affect gene function. In the context of the growing literature on amino acid depletion diets and related disorders^41–47^ (for example, cancer, longevity, stemness, autophagy and Charcot–Marie–Tooth (CMT) disease), substitutants may have a profound effect on cell function.

## Methods

### Cell-culture and reagents

MD55A3 melanoma cells were derived from metastatic melanoma tumour resections^6^, collected with informed patient consent under a protocol approved by the National Institutes of Health (NIH) IRB Ethics Committee and approved by the MD Anderson IRB (protocol numbers 2012-0846, LAB00-063 and 2004-0069; NCT00338377). MD55A3 were cultured in Roswell Park Memorial Institute 1640 Medium (RPMI 1640, Gibco) supplemented with 10% heat-inactivated fetal bovine serum (Sigma), 25 mM HEPES (Gibco) and 100 U ml^−1^ penicillin/streptomycin. All other cell lines were purchased for ATCC. hTERT RPE-1, MCF-7, MDA-MB-231, HEK 293T and HT29 were cultured in in Dulbecco’s modified Eagle’s medium (DMEM, Gibco), supplemented with 10% fetal bovine serum and 100 U ml^−1^ penicillin/streptomycin; MCF10A cells were cultured in DMEM/F12 containing HEPES (Gibco) supplemented with 5% horse serum (Gibco), EGF (10 ng ml^−1^; Millipore), insulin (10 μg ml^−1^; Sigma) and hydrocortisone (500 ng ml^−1^; Sigma). HCT116 cells were cultures in RPMI 1640 medium (Gibco) supplemented with 10% fetal bovine serum and 100 U ml^−1^ penicillin/streptomycin. All cell lines were maintained in a humidified atmosphere containing 5% of CO_2_ at 37 °C. All cell lines were tested regularly by PCR for mycoplasma contamination and were found to be negative.

Tryptophan-free DMEM/F12 medium was purchased from US Biologicals. Tyrosine-free and phenylalanine-free DMEM were custom-made (Cell Culture Technology). All these media were supplemented with 10% heat-inactivated dialysed fetal bovine serum (Gibco) and 100 U ml^−1^ penicillin/streptomycin. IFNγ (PeproTech) was used at 250 U ml^−1^ for 48 h. MG-132 (Selleckchem), dissolved in DMSO, was used at a final concentration of 10 µM. The IDO inhibitor 1-methyl-l-tryptophan (Sigma) was dissolved in 0.1N NaOH at a 20 mM concentration, adjusted to pH 7.5, filter sterilized and used at a final concentration of 300 µM. Polyethylenimine (PEI, Polysciences) was dissolved in water at a concentration of 1 mg ml^−1^.

### Generation of reporter plasmids

TurboGFP was amplified by PCR using the primers listed in Supplementary Table 10 and the pLKO.1-tGFP plasmid (kind gift from R. Beijersbergen) as a template. The resulting PCR product was cloned into pCDH-blast vector by restriction–ligation cloning into the XbaI and NotI sites.

Mutagenesis was performed using the GeneArt site-directed mutagenesis system (Invitrogen) according to the manufacturer’s instructions. The primers used for generating tGFP F26W and F26A are listed in the Supplementary Table 10. Mutagenesis was performed on the pCDH-Blast-tGFP plasmid.

V5-ATF4^1–63/W93Y^-tGFP was generated by PCR, where the codon for tryptophan (codon 93) was replaced by a codon for tyrosine in the original V5-ATF4^1–63^-tGFP sequence. A first PCR was performed to amplify V5-ATF4 using the primers listed in Supplementary Table 10. This resulting PCR product was extended with tGFP by a second PCR with the V5-ATF4^1–63^-tGFP plasmid as a template. The V5-ATF4^1–63/W93Y^-tGFP gene was then inserted in the pCDH-Blast vector by restriction/ligation cloning in the XbaI and NotI sites.

A DNA sequence coding for the amino acid sequence LEQLESIINFEKL, or the mutated forms thereof, was cloned immediately downstream of the tGFP sequence in the pCDH-V5-ATF4^1–63/W93Y^-tGFP reporter constructs. This was done by PCR on the V5-ATF4^1–63/W93Y^-tGFP construct as template and using the primers listed in Supplementary Table 10. The resulting PCR products were then inserted by restriction–ligation cloning in the XbaI and NotI sites in the pCDH-Blast vector.

The H2-Kb gene was amplified from cDNA using the primers in Supplementary Table 10. The PCR product was cloned into the pCDH-puro backbone by restriction/ligation cloning by making use of the XbaI and EcoRI sites. Next, the puromycin selection cassette was replaced by a hygromycin cassette. This cassette and the PGK promoter were amplified by PCR using the primers in Supplementary Table 10 and the pLenti-Hygro plasmid as a template. The resulting DNA fragment was introduced between the BamHI and XhoI sites of the pCDH-H2-Kb plasmid by a restriction/ligation procedure.

All resulting plasmids were sequence verified by Sanger sequencing (Macrogen).

### Lentiviral production and transduction

For lentivirus production, 4 × 10^6^ HEK 293T cells were seeded per 100 mm dish, one day prior to transfection. For each transfection, 10 µg of the pCDH vector of interest, 5 µg of pMDL RRE, 3.5 µg pVSV-G AND 2.5 µg of pRSV-REV plasmids were mixed in 500 µl of serum-free DMEM. Next, 500 µl of serum-free DMEM containing 63 µl of a 1 mg ml^−1^ PEI solution was added. The entire mix was vortexed and left for 15 min at room temperature after which it was added to the HEK 293T cells to be transfected. The next day, the medium was replaced and the lentivirus-containing supernatants were collected 48 and 72 h post transfection, and snap frozen in liquid nitrogen. Target cells were transduced by supplementation of the lentiviral supernatant with 8 µg ml^−1^ polybrene (Sigma). One day after transduction, the transduced cells were selected by addition of 5 µg ml^−1^ blasticidin (Invivogen) or 50–1000 µg ml^−1^ hygromycin B (Gibco) to the medium.

### Amino acid mass spectrometry

Cells were washed with cold PBS and lysed with lysis buffer composed of methanol/acetonitrile/H_2_O (2:2:1). The lysates were collected and centrifuged at 16,000*g* (4 °C) for 15 min and the supernatant was transferred to a new tube for liquid–chromatography mass spectrometry (LC–MS) analysis. For media samples, 10 µl of medium was mixed with 1 ml lysis buffer and processed as above.

LC–MS analysis was performed on an Exactive mass spectrometer (Thermo Scientific) coupled to a Dionex Ultimate 3000 autosampler and pump (Thermo Scientific). Metabolites were separated using a Sequant ZIC-pHILIC column (2.1 × 150 mm, 5 µm, guard column 2.1 × 20 mm, 5 µm; Merck) using a linear gradient of acetonitrile (A) and eluent B (20 mM (NH_4_)_2_CO_3_, 0.1% NH_4_OH in ULC/MS grade water (Biosolve), with a flow rate of 150 µl min^−1^. The mass spectrometer was operated in polarity-switching mode with spray voltages of 4.5 kV and −3.5 kV. Metabolites were identified on the basis of exact mass within 5 ppm and further validated by concordance with retention times of standards. Quantification was based on peak area using LCquan software (Thermo Scientific).

### Public datasets

The following publicly available datasets were used for this study: Proteomics Data Commons PDC000234, PDC000270, PDC000198, PDC000221, PDC000173, PDC000204, PDC000110, PDC000116, PDC000153 and PDC000303; and PRIDE datasets (PXD020079, PXD020224 and PXD022707). The UNIPROT Database is sourced from UNIPROT.org with the identifier UP000005640.

### Analysis of immunoprecipitation-based mass spectrometry data

#### Data generation

At the end of each experiment intended for V5-tag pulldown, cells were treated with 10 µM MG-132 for 4 h and subsequently collected by trypsinization and centrifugation. Next, cells were lysed in 300 µl ELB lysis buffer (50 mM HEPES, 125 mM NaCl, 0.5% (v/v) Tween-20 and 0.1% (v/v) Nonidet P40 substitute. Next, 3 µl mouse anti-V5 antibody solution (1.0 mg ml^−1^, Invitrogen) was added to the lysate and the samples were incubated on a rotating wheel at 4 °C overnight. Pulldowns were performed with Dynabeads protein G (Invitrogen) according to manufacturer’s protocol. All pulled down protein was eluted in 30 µl of 1× Laemmli buffer.

Next, the eluates were run briefly into a 4–12% Criterion XT Bis-Tris gel (Bio-Rad) and short, Coomassie-stained gel lanes were excised for each sample. Proteins were reduced with 6.5 mM DTT, alkylated with 54 mM iodoacetamide and digested in-gel with trypsin (Gold, mass spectrometry grade, Promega, 3 ng μl^−1^) overnight at 37 °C. Extracted peptides were vacuum dried, reconstituted in 10% formic acid and analysed by nanoLC–MS/MS on an Orbitrap Fusion Tribrid mass spectrometer equipped with a Proxeon nLC1000 system. Peptides were loaded directly on the analytical column and separated in a 90-min gradient containing a non-linear increase from 5% to 26% solvent B.

#### Generation of search database

Five search databases were generated, to cover all possibilities of aberrant protein production from the V5–ATF4^1–63^–tGFP reporter protein. The first database consisted of the original ATF4 in-frame protein sequence, the ATF4 sequence until W93 and frame-shifted (+1) at W codon until the first stop codon (Fig. 1a), the in-frame ATF4 protein sequence with the tryptophan replaced by every other amino acid, and the ATF4 protein sequences where the tryptophan codon is skipped. The second database consisted of the tryptic peptide spanning the tryptophan codon in the in-frame ATF4 sequence, and was generated by replacing every amino acid in the sequence to every other possible amino acid. The third database was generated by replacing every phenylalanine in the in-frame ATF4 sequence to every other amino acid. The fourth database was generated by replacing every tyrosine in the in-frame ATF4 sequence to every other amino acid. Finally, Translational bypass was checked for with a final database consisting of the tryptic peptides that would originate from exclusion of W93. And additionally, the peptide that would arise from the event where a ribosome taking off with tRNA-Glu-TTC loaded in the P-site, and re-starting translation at the next GAA codon.

#### Searching of immunoprecipitation–mass spectrometry data against the databases

The search was performed using MaxQuant (version 1.6.0.16)^48^. Peptide false discovery rate (FDR) threshold was set at 0.01. The parameters of the search were optimized for increasing sensitivity and were deposited in the PRIDE database^49^.

### Analysis of 2D proteomics data

#### Data generation

MD55A3 and MCF10A expressing the V5–ATF4^1–63^–tGFP^+1^ reporter were used for this purpose. On the first day, cells were seeded in 15cm dishes at around 60% confluency. The next day, cells were rinsed with PBS and were exposed to the appropriate treatment (IFNy or tryptophan-free medium). As control, tryptophan-free medium was supplemented with 5μg ml^−1^ l-tryptophan (Sigma). After 48 h of treatment, 10 μM MG-132 was added directly in the plates and cells were incubated for 4 h at 37 °C. Then, cells were washed once with PBS and collected by trypsinization and centrifugation. Cell pellets were washed once with PBS, after which the cell pellet was snap-frozen in liquid nitrogen.

Then, the samples were reduced and alkylated in heated guanidine (GuHCl) lysis buffer as described^50^. After dilution to 2M GuHCl, proteins were digested twice (4h and overnight) with trypsin (Sigma) at 37 °C, enzyme/substrate ratio 1:50. Digestion was quenched by the addition of TFA (final concentration 1%), after which the peptides were desalted on a Sep-Pak C18 cartridge (Waters). Samples were vacuum dried and stored at −80 °C until fractionation.

Dried digests were subjected to basic reversed-phase (HpH-RP) high-performance liquid chromatography for offline peptide fractionation. Two-hundred-and-fifty micrograms of peptides were reconstituted in 95% 10 mM ammonium hydroxide (NH_4_OH, solvent A)/5% (90% acetonitrile (ACN)/10mM NH_4_OH, solvent B) and loaded onto a Phenomenex Kinetex EVO C18 analytical column (150 mm × 2.1 mm, particle size 5 μm, 100 Å pores) coupled to an Agilent 1260 HPLC system equipped with a fraction collector. Peptides were eluted at a constant flow of 100 μl min^−1^ in a 90-min gradient containing a nonlinear increase from 5–30% solvent B. Fractions were collected and concatenated to 24 fractions per sample replicate. All fractions were analyzed by nanoLC–MS/MS on an Orbitrap Fusion Tribrid mass spectrometer equipped with an Easy-nLC1000 system (Thermo Scientific). Peptides were directly loaded onto the analytical column (ReproSil-Pur 120 C18-AQ, 1.9 μm, 75 μm × 500 mm, packed in-house). Solvent A was 0.1% formic acid/water and solvent B was 0.1% formic acid/80% acetonitrile. Samples were eluted from the analytical column at a constant flow of 250 nl min^−1^ in a 2 h gradient containing a linear increase from 8–32% solvent B. Mass spectrometry settings were as follows: full MS scans (375–1500 *m*/*z*) were acquired at 60,000 resolution with an automatic gain control (AGC) target of 3 × 10^6^ charges and max injection time of 45 ms. Loop count was set to 20 and only precursors with charge state 2–7 were sampled for MS2 using 15,000 resolution, MS2 isolation window of 1.4 *m*/*z*, 1 × 10^5^ AGC target, a max injection time of 22 ms and a normalized collision energy of 26.

#### Generation of mutant database

The human proteome was downloaded from UNIPROT^51^. All instances of tryptophan were replaced by other amino acids in a separate database (FASTA file).

#### Database Search and filtering

Each proteomics dataset was searched against the database. The parameters of the search are deposited in the PRIDE database^49^. Peptide FDR threshold was set at 0.01. After the search, only the tryptic peptides spanning the endogenous tryptophan codon were retained and used for further analysis. Further filtering was done to keep only the reproducibly detected peptides.

### Analysis of immunopeptidomics data

#### Data acquisition

Immunopeptidomics data of colorectal cancer was sourced from the published study^39^. In addition, for glioblastoma (RA) datasets- HLA bound peptides were eluted as previously described^52^ from 200 million RA cells treatment or not with 500 IU ml^−1^ IFNγ for 48 h in 4 biological replicates each condition. W6/32 antibody cross linked to protein-A sepharose 4B beads was used for the immunoaffinity purification. HLA-bound peptides were measured on a LC-MS/MS system consisted of an Easy-nLC 1200 connected to a Q Exactive HF-X mass spectrometer (Thermo Fisher Scientific) as previously described^52^. Peptides were separated with a flow rate of 250 nl min^−1^ by a gradient of 0.1% formic acid (FA) in 95% ACN and 0.1% FA in water. Full MS spectra were acquired in the Orbitrap from *m*/*z* = 300–1,650 with a resolution of 60,000 (*m*/*z* = 200), ion accumulation time of 80 ms and AGC of 3 × 10^6^ ions. MS/MS spectra were acquired in a data dependent manner on the twenty most abundant precursor ions with a resolution of 30,000 (*m*/*z* = 200), an ion accumulation time of 120 ms, isolation window of 1.2 *m*/*z*, AGC of 2 × 10^5^ ions, dynamic exclusion of 20 s, and a normalized collision energy (NCE) of 27 was used for fragmentation. The peptide match option was disabled.

#### Generation of mutant database, search and filtering

The human proteome was downloaded from UNIPROT^51^ (release-2011_01, downloaded June 2019). All instances of tryptophan were replaced by phenylalanine and stored in a DB (FASTA file). The RAW MS data files were analysed using MaxQuant (version 1.6.0.16) by performing a search against the generated database. The parameter file for the search is deposited in the PRIDE Database and basic parameters^19^ are provided as Supplementary Table 11. Briefly, we performed search scans with FDR 0.01, 0.05 and 0.1, and for lower thresholds (FDR < 0.1) controlled with other tryptophan substitutants. Additionally, we validated the peptides using targeted mass-spectrometry analysis (see methods, Supplementary Table 9). Only the fragmented peptides spanning the tryptophan codon were retained for further analysis of substitutant peptides. Further filtering was done to keep only the reproducibly detected peptides.

#### Validation with parallel reaction monitoring

Peptides were ordered from ThermoFisher Scientific as crude (PePotec grade 3) with amino acid where heavy stable isotope atoms were incorporated for parallel reaction monitoring. Synthetic peptides were spiked into the peptidomic samples at a concentration of 1 pmol μl^−1^. The mass spectrometer was operated at a resolution of 120,000 (at *m*/*z* = 200) for the MS1 full scan, with an ion injection time of 80 ms, AGC of 3 × 10^6^ and scanning a mass range from 300 to 1,650 *m*/*z*. Each peptide was isolated with an isolation window of 1.2 *m*/*z* prior to ion activation by high-energy collision dissociation (HCD, NCE = 27). Targeted MS/MS spectra were acquired at a resolution of 30,000 (at *m*/*z* = 200) with 80 ms ion injection time and an AGC of 5 × 10^5^.

The PRM data were processed and analysed as previously described^52^ using Skyline (v4.1.0.18169)^53^. Ion mass tolerance of 0.05 *m*/*z* was used to extract fragment ion chromatograms and peak lists for the heavy-labelled peptides and endogenous light counterparts were extracted. MS/MS matching assessment was performed by pLabel (v2.4.0.8, pFind studio, Sci. Ac.) and Skyline (MacCoss Lab, v21.1.0.146).

### Structural analysis

The structural implications of the W>F substitutions were analysed using the HOPE meta-server^54^ which creates human-readable reports describing the structural and functional importance of the substituted residue, for example, from known variants and mutation data stored in the UniprotKB^55^ and sequence variability data from large-scale multiple sequence alignments in the HSSP databank^56^. If a suitable template structure model is available in the Protein Data Bank^57^, HOPE also creates homology models of the wildtype protein structures. All created homology models were visually inspected in Coot to assess whether the tryptophan residues made structurally important hydrogen bonds through their side-chains that are lost by W>F substitutions. It should be noted that possible hydrogen bonds with other proteins cannot be studied from these homology models.

### Analysis of large-scale proteomics data of human cancer

LSCC, BC, LUAD, CCRCC, HCC, HNSCC, PDA, GBM, OV and BC-PDX data were download from the Proteomics Data Center^22^ in MZML file format. The human proteome was downloaded from UNIPROT^51^ (release-2011_01, downloaded June 2019), and all instances of tryptophan amino-acids in the proteome were changed to all other amino acids except Lysine and Arginine, in order to avoid creation of tryptic cleavage site in the scan. The resultant FASTA file was used as Philosopher pipeline^58^ was used to detect all peptides in mass-spectrometry datasets (MZML files), including the substitutant peptides. Briefly, MSFragger^59^ was used for peptide detection with the following parameters; Precursor mass lower: −20 ppm, Precursor mass upper: 20 ppm, precursor mass tolerance: 20 ppm, calibrate mass: True, Deisotoping: True, mass offset: False, isotope error: Standard, digestion: Strictly tryptic (Max. missed cleavage: 2), Variable modifications (For iTRAQ datasets): 15.99490 M 3, 42.01060 [^ 1, 229.162932 n^ 1, 229.162932 S 1, Variable modifications (For TMT datasets): 15.99490 M 3, 42.01060 [^ 1, 144.1021 n^ 1, 144.1021 S 1, Min Length: 7, Max Length: 50, digest mass range: 500:5000 Daltons, Max Charge: 2, remove precursor range: −1.5, 1.5, topN peaks: 300, minimum peaks: 15, precursor range: 1:6, add Cysteine: 57.021464, add Lysine (for ITRAQ datasets): 144.1021, add Lysine (for TMT datasets): 229.162932,among other basic parameters (PARAMETER.yml is submitted in the as Supplementary Table 12). PeptideProphet^60^ was then used for Peptide Validation with following parameters (accmass: TRUE, decoyprobs: TRUE, expectScore: TRUE, Glycosylation: FALSE, ICAT: FALSE, masswidth: 5, minimum probability after first pass of a peptide: 0.9, minimum number of NTT in a peptide: 2, among other parameters (Supplementary Table 12). Next, isobaric quantification was next undertaken separately for TMT and iTRAQ datasets with following parameters (bestPSM: TRUE, level: 2, minProb 0.7, ion purity cut-off: 0.5, tolerance: 20 ppm, among other parameters (Supplementary Table 12). Thereafter, FDR filtering was implemented to retain only confident peptides with following parameters (FDR < 0.01, peptideProbability: 0.7, among other parameters (Supplementary Table 12). Thereafter, TMT-integrator^58^ was used to integrate isobaric quantification with following parameters (retention time normalization: False, minimum peptide probability on top of FDR filtering (TMT datasets): 0.9, minimum peptide probability on top of FDR filtering (for iTRAQ dataset): 0.5, among other parameters). Substitutant peptides were fetched from the reports of TMT integrator command, and any detected peptide intensity score for a sample normalized to the reference channel above 0 (log scale) was considered as a positive peptide for that sample using a R-script. R was used to plot density plots as well as Barplots for number of peptide detections (Fig. 3f, Extended Data Fig. 3). For all intra-tumour type analysis, a filter for maximum number of samples (vertical lines in Extended Data Fig. 3) was applied to retain peptides with higher specificity in expression. Next, protein expression profiles for each cancer type were downloaded in already analysed format from PDC commons (https://pdc.cancer.gov). PERL scripts were designed to count number of substitutants when a gene is lowly expressed (intensity < 0) or highly expressed (intensity > 0). Gene ontology (GO)-term enrichment analysis was done using ToppGene^61^. Phosphoproteome data was downloaded from PDC commons (https://pdc.cancer.gov), and a similar analysis as to proteome analysis was undertaken using a customized PERL script.

### Western blotting

Straight lysates from cells were made in 6 wells by addition of 200 µl of 1× Laemmli buffer. All protein samples were run on SDS-PAGE gels and blotted on 22 µm pore size nitrocellulose membranes (Santa Cruz). V5 stainings were performed using V5 tag monoclonal antibodies (Invitrogen, R960-25; 1:1,000), tGFP staining with rabbit anti TurboGFP (Invitrogen, PA5-22688; 1:1,000), IDO1 was visualized with rabbit anti-IDO D5J4E (Cell Signaling, 86630, 1:1,000) and tubulin with anti-tubulin (DM1A, Sigma, 1:10,000).

Subsequent stainings were performed with IRDye 680RD donkey anti-mouse (LI-COR, 926-68072, 1:10,000) and IRDye 800CW goat anti-rabbit (LI-COR, 926-32211, 1:10,000) secondary antibodies. Visualization was performed by use of an Odyssey infrared scanning device (LI-COR).

### WARS1 activity assay

The human WARS1 gene was cloned in the LIC1_1 vector by PCR amplification and ligation independent cloning using the following primers: cagggacccggtATGCCCAACAGTGAGCCCGCATCTCTGC and cgaggagaagcccggttaCTGAAAGTCGAAGGACAGCTTCCGGGGAG. The inserted sequence was verified by Sanger sequencing and the recombinant protein was expressed in Rosetta2(DE3) cells. In short, cells were grown at 37 °C until OD600 of 0.7. Next, protein expression was induced by addition of 0.4 mM IPTG and the cells were grown overnight at 18 °C. After lysis, the recombinant WARS1 protein was purified using nickel beads, after which the protein was reconstituted in 25 mM Tris pH 8.0, 200 mM NaCl and 1 mM TCEP.

WARS1 aminoacylation activity toward different amino acids was estimated by measuring released phosphate. The assay was performed in a 50 µl reaction volume containing 20 µM purified WARS1 enzyme, 100 mM TRIS, 10 mM MgCl_2_, 40 mM KCL, 1 mM dithiothreitol, 0.25 U µl^−1^ pyrophosphatase (Sigma-Aldrich) and 0.5 mM of tryptophan, serine, glycine, phenylalanine or methionine. The reaction mixture was incubated at 37 °C for 30 min. Afterwards, 100 µl of BIOMOL Green TM (Enzo Life Sciences) was added and the samples were incubated at room temperature for 30 min. The released phosphate was quantified by measuring absorbance at 620 nm with an Infinite 200 microplate reader (Tecan).

### Fluorescence-activated cell sorting

#### Measurement of tGFP fluorescent intensity

Cells expressing the tGFP reporters were seeded, and treatment was started the next day. 48 h after the start of treatment, the cells were collected by trypsinization and centrifugation. Next, the cells were analysed on an Attune NxT machine (Thermo Fisher Scientific) using Attune Nxt software version 4.2 and the data were analysed using FlowJo V10 software (FlowJo).

#### Measurement of H2-Kb-bound SIINFEKL levels

MD55A3 and HT29 cells were transduced with lentiviruses produced from pCDH-Hygro-H2-Kb and selected with hygromycin (Invitrogen)^8^. Next, the H2-Kb expressing cells were transduced with lentiviruses generated from the pCDH-V5-ATF4^1–63/W93Y^-tGFP-SIINFEKL or the mutant versions thereof. Transduced cells were selected for using 5 µg ml^−1^ blasticidin (Invivogen).

For the detection of presented H2-Kb-bound SIINFEKL peptides, cells were treated for 48 h with 250 U ml^−1^ IFNγ (Peprotech), 1MT (IDOi, 300 μM, Sigma) and/or tryptophan-less DMEM/F12 (USBiologicals). Then, cells were washed with PBS and detached using PBS–EDTA (50 µM). Next, cells were pelleted and washed with PBS/0.5% BSA and incubated with APC anti-mouse H2-Kb-bound to SIINFEKL antibodies (Biolegend, clone 25-D1.16, 141606; 1:200 in PBS/0.1% BSA) for 30 min on ice, in the dark. The cells were then washed twice with PBS-BSA and analysed on an Attune NxT machine using Attune Nxt software version 4.2 (Thermo Fisher Scientific). Data were analysed using FlowJo V10 software (FlowJo). HT29 H2-Kb- and ATF4^1–63^(W93Y)–tGFP–SIINwEKL-expressing cells contained a highly variable signal for H2-Kb-bound SIINFEKL after treatment, the highly positive cells were sorted out. First, these cells were treated for 48 h with IFNγ, after which they were stained for H2-Kb bound to SIINFEKL as described above. The top 7.5% positive cells were sorted out of the population using a BD FACSAria Fusion machine (BD biosciences).

#### OT-I T cell SIINFEKL recognition assays

OT-I (B6J) mice were originally from The Jackson Laboratory. Mice used for experiments were between 3 and 12 weeks old and of both sexes. All experiments involving animals were performed in accordance with Dutch and European regulations on care and protection of laboratory animals and have been approved by the local animal experiment committee at Netherlands Cancer Institute, DEC NKI (OZP ID 12051). Mice were bred and maintained in accordance with institutional, national and European guidelines for Animal Care and Use.

OT-I T cells were isolated using Dynabeads Untouched Mouse CD8 Cells Kit (Invitrogen) according to the manufacturer’s protocol. T cells were initially maintained in Roswell Park Memorial Institute 1640 Medium (Gibco) containing 10% fetal bovine serum (Sigma), 50 µM 2-mercaptoethanol (Sigma), 100 U ml^−1^ penicillin, 100 μg ml^−1^ streptomycin (both Gibco), 100µg/mL IL-2 (ImmunoTools),5 µg/mL IL-7 (ImmunoTools) and 10 µg ml^−1^ IL-15 (ImmunoTools).

MD55A3 cells expressing H2-Kb and V5–ATF4^1–63^(W93Y)–tGFP–SIINFEKL or V5–ATF4^1–63^(W93Y)–tGFP–SIINwEKL were treated for 2 days with the indicated treatments. To the IFN-treated samples, 7.2 × 10^2^ µg ml^−1^ purified PEG–His–*mp*Kynureninase^38^ and 2 µM pyridoxal 5′-phosphate hydrate (Sigma) were added. At the end of the treatment, the cancer cells were detached using PBS-EDTA and seeded at 100,000 cells per well in a 96 U-shaped-well plate. Next, 100,000 OT-I T cells were added to start the co-culture and the solution was supplemented with BD Golgiplug (BD Biosciences). The co-culture samples were then incubated for 12 h at 37 °C in a humidified CO_2_ incubator.

HT29 cells expressing H2-Kb or H2-Kb and V5–ATF4^1–63^(W93Y)–tGFP–SIINwEKL were treated for two days with the indicated treatments. To the IFN-treated samples, 36 µg ml^−1^ purified His–*mp*Kynureninase and 2 µM pyridoxal 5′-phosphate hydrate (Sigma) were added. In one of the IFN-treated samples 1MT (IDOi, 300 μM, Sigma) was added. At the end of the treatment, the cancer cells were detached using PBS/EDTA and seeded at 100,000 cells per well in a U-shaped 96 well plate. Next, 100,000 OT-I T cells were added to start the co-culture and the solution was supplemented with BD Golgiplug (BD Biosciences). The co-culture samples were then incubated for 4h at 37 °C in a humidified CO_2_ incubator.

Next, the cells were pelleted by centrifugation, blocked with 0.1% PBS-BSA and stained with anti-mouse CD8-VioBlue antibodies (Miltenyi, 130-111-638, 1:100) and Live/Dead Fixable near-IR dead cell stain kit (Invitrogen). Subsequently, the cells were fixed and permeabilized using the eBioscience Foxp3 Transcription Factor Staining Buffer Set (Invitrogen) according to manufacturer’s instructions. Next, the cells were stained with APC-conjugated anti-mouse IFNγ (Miltenyi, 130-109-723 and 130-120-805, 1:100) and PE-conjugated anti-mouse TNF (Miltenyi, 130-109-719 and 130-102-386, 1:100) antibodies. Cells were then washed and analysed on a BD LSR Fortessa (BD Biosciences). The data were analysed using FlowJo V10 software (FlowJo).

### OT-I T cell-mediated killing assay

HT29 H2-Kb- or H2-Kb and ATF4^1–63^(W93Y)–tGFP–SIINwEKL-expressing cells were mock treated for 48 h or treated with IFNγ in tryptophan-less DMEM/F12 medium in 12 well plates. To the IFNγ-treated samples, 36 µg ml^−1^ purified His–*mp*Kynureninase and 2 µM pyridoxal 5′-phosphate hydrate (PLP, Sigma) were added. After this treatment, the medium was replaced with fresh DMEM supplemented with kynureninase and PLP for the corresponding samples. Then OT-I cells were added in ratios HT29:OT-I of 4:1, 2:1 and 1:1. The co-cultures were left for 24 h at 37 °C in a humidified CO_2_ incubator. After the co-culture, the cells were fixed using 4% formaldehyde (Merck) in PBS. Then the cells were stained using crystal violet (0.1% in water) for 30 min, after which the plates were washed thoroughly in water and left to dry. Bound crystal violet was extracted using a 10% acetic acid solution (in water). To quantify the bound crystal violet in each well, the solution from the well was diluted tenfold with water and the absorbance was measured at 590 nM using an Infinite 200 PRO reader (Tecan).

### Kynureninase activity measurement

Kynurenine (l-kyn) analysis: Samples (50 µl) were mixed with 50 ul l-kyn-d4 (1 µM) in water and 10 µl of trifluoro acetic acid. Mixtures were centrifuged (10 min, 20,000*g*, 4’C). Supernatant (50 µl) was diluted with water (200 µl) and 10 µl was analysed by LC–MS using a API4000 (Sciex). Separation was achieved using a Zorbax Extend C18 column 100 × 2 mm ID) and an isokratic mobile phase comprising 0.1% formic acid in water: methanol (98:2 v/v). MS detection by multiple reaction monitoring using ion pairs 209.3/192.1 (l-kyn) and 213.3/196.1 (l-kyn-d4).

### Induction of T cells reactive to substitutant peptides

PBMCs were isolated from buffy coats from previously HLA-typed healthy donor buffy coats from Oslo University Hospital Blood Bank. The study was approved by the Regional Ethics Committee (REC) and informed consent was obtained from healthy donors in accordance with the declaration of Helsinki and institutional guidelines (REC 2018/2006 and 2018/879). Isolation of T cells reactive to substitutant peptides was performed as previously described^6,40^ with modifications. In brief, on day −4 monocytes were isolated from PBMCs of HLA-A*24:02 positive healthy donors using CD14-reactive microbeads and an AutoMACS Pro Separator (Miltenyi Biotec). Cells were then cultured for three days in CellGro GMP DC medium (CellGenix) supplemented with 1% (v/v) human serum (HS, Trina Biotech) and 1% (v/v) penicillin–streptomycin containing 10 ng ml^−1^ IL-4 (PeproTec) and 800 IU ml^−1^ GM-CSF (Genzyme). Subsequently, monocyte-derived-dendritic cells were matured for 14–16 h by supplementing cultures with 800 IU ml^−1^ GM-CSF, 10 ng ml^−1^ IL-4, 10 ng ml^−1^ lipopolysaccharide (LPS; Sigma-Aldrich) and 5 ng/ml IFNγ (PeproTech). On day −1, autologous naive CD8^+^ T cells were isolated using a CD8^+^ T cell isolation kit and AutoMACS Pro Separator (Miltenyi Biotec). Naive CD8^+^ T cells were cultured overnight in TexMACS medium (Miltenyi Biotec) supplemented with 1% (v/v) penicillin/streptomycin and 5 ng ml^−1^ IL-7 (PeproTech). On day 0, monocyte-derived dendritic cells were peptide-pulsed with individual substitutant peptides for 2 h at a concentration of 1 μg ml^−1^, or incubated with DMSO vehicle. A total of six substitutant peptides (BI1, KLHL4, W2PPT5, F5GXS0, F8VXG7 and G5E9G0) were included. Individually peptide-loaded monocyte-derived dendritic cells were collected and pooled before combining with naive T cells for co-culture in CellGro GMP DC medium supplemented with 5% human serum and 30 ng ml^−1^ IL-21 (PeproTech) at a DC:T cell ratio of 1:2. In parallel control cultures, naive T cells were co-cultured with DMSO-vehicle-treated monocyte-derived dendritic cells. On days 3, 5 and 7, half of the medium was removed and replaced with fresh medium supplemented with 10 ng ml^−1^ of both IL-7 and IL-15 (PeproTech). On day 10, co-cultures were screened for the presence of substitutant pMHC multimer-reactive CD8^+^ T cells. pMHC multimers conjugated to four different streptavidin (SA)–fluorochrome conjugates were prepared in-house as previously described^62,63^. SA–phycoerythrin (SA–PE), SA–phycoerythrin-CF594 (SA-PE-CF594), SA–allophycocyanin (SA–APC) and SA–Brilliant Violet 605 (SA–BV605). Each pMHC multimer was labelled with two different fluorochromes for increased specificity. Positive T cells were identified by Boolean gating strategy in FlowJo (TreeStar) v10.6.2 software as live CD8^+^ T cells staining positively for two pMHC multimer fluorochromes and negatively for the two other pMHC multimer fluorochromes, shown in Supplementary Fig. 3 and as previously described^64^.

### Reporting summary

Further information on research design is available in the Nature Research Reporting Summary linked to this paper.

## Online content

Any methods, additional references, Nature Research reporting summaries, source data, extended data, supplementary information, acknowledgements, peer review information; details of author contributions and competing interests; and statements of data and code availability are available at 10.1038/s41586-022-04499-2.

## Supplementary information

Supplementary Figures Supplementary Figs. 1–3, including the uncropped blots and gating strategy.

Reporting Summary

Supplementary Tables Supplementary Tables 1–12.

## Data Availability

The mass spectrometry proteomics data have been deposited to the ProteomeXchange Consortium via the PRIDE^49^ partner repository with the dataset identifier PXD028921.

## Code availability

The scripts used in this study are available with additional details at https://github.com/apataskar/substitutants_manuscript.
