# Author Correction: Tryptophan depletion results in tryptophan-to-phenylalanine substitutants

**DOI:** 10.1038/s41586-022-05097-y

**Published:** 2022-07-15

**Authors:** Abhijeet Pataskar, Julien Champagne, Remco Nagel, Juliana Kenski, Maarja Laos, Justine Michaux, Hui Song Pak, Onno B. Bleijerveld, Kelly Mordente, Jasmine Montenegro Navarro, Naomi Blommaert, Morten M. Nielsen, Domenica Lovecchio, Everett Stone, George Georgiou, Mark C. de Gooijer, Olaf van Tellingen, Maarten Altelaar, Robbie P. Joosten, Anastassis Perrakis, Johanna Olweus, Michal Bassani-Sternberg, Daniel S. Peeper, Reuven Agami

**Affiliations:** 1 Division of Oncogenomics, Oncode institute, The Netherlands Cancer Institute, Amsterdam, The Netherlands; 2 Division of Molecular Oncology and Immunology, Oncode institute, The Netherlands Cancer Institute, Amsterdam, The Netherlands; 3 Department of Cancer Immunology, Institute for Cancer Research, Oslo University Hospital Radiumhospitalet, Oslo, Norway; 4 Institute of Clinical Medicine, University of Oslo, Oslo, Norway; 5 Lausanne Branch, Ludwig Institute for Cancer Research, Lausanne University Hospital (CHUV) and University of Lausanne (UNIL), Lausanne, Switzerland; 6 Center of Experimental Therapeutics, Department of Oncology, Lausanne University Hospital (CHUV), Lausanne, Switzerland; 7 NKI Proteomics facility, The Netherlands Cancer Institute, Amsterdam, The Netherlands; 8 Department of Molecular Biosciences, University of Texas, Austin, TX USA; 9 Division of Pharmacology, The Netherlands Cancer Institute, Amsterdam, The Netherlands; 10 Biomolecular Mass Spectrometry and Proteomics, Bijvoet Center for Biomolecular Research, Utrecht Institute for Pharmaceutical Sciences, Utrecht University and Netherlands Proteomics Centre, Utrecht, The Netherlands; 11 Division of Biochemistry, The Netherlands Cancer Institute, Amsterdam, The Netherlands; 12 Erasmus MC, Department of Genetics, Rotterdam University, Rotterdam, The Netherlands

**Keywords:** Tumour immunology, Proteomics

Correction to: *Nature* 10.1038/s41586-022-04499-2Published online 9 March 2022

In the version of this article initially published, the Acknowledgements statement for Johanna Olweus did not include thanks for support to “The Norwegian Cancer Society, The Research Council of Norway and European Research Council (ERC) under the European Union’s Horizon 2020 research and innovation programme (grant agreement no. 865805),” which has now been amended in the HTML and PDF versions of the article.

In addition, we wish to clarify the filters used in the analysis of LSCC cancer proteomes (Fig. 3a and Fig. 3f). As described in the Methods section “Analysis of large-scale proteomics data of human cancer,” for intra-tumour type analysis (Fig. 3f), a filter for maximum number of samples was applied to retain peptides with higher specificity in expression. However, we would like to add that in the tumor-specific analysis (Fig. 3a) this filter was not applied for W>F substitutants both because of their exclusive significant and specific distribution (Extended Data Fig. 3, p.val 1.13E–09) and in order to optimize inclusion of signal for gene expression correlation analysis (Fig. 3c–e). Importantly, both filtering strategies indicated a similar biological conclusion, as reported in the manuscript (Fig. 3a and Fig. 3f). We apologize for this unclarity and thank Gautam Kok, Imre Schene and Sabine Fuchs for bringing it to our attention.

