# Supplementary material for: Tryptophan depletion results in tryptophan-to-phenylalanine substitutants
Source: Nature. 2022 Mar 9;603(7902):721–7. doi: 10.1038/s41586-022-04499-2 (PMC8942854; doi:10.1038/s41586-022-04499-2)
Supplement: Supplementary file 1 — Supplementary Figs. 1–3, including the uncropped blots and gating strategy. [file 41586_2022_4499_MOESM1_ESM.pdf]

nature portfolio

https://doi.org/10.1038/s41586-022-04499-2

# Supplementary information

# Tryptophan depletion results in tryptophan-to-phenylalanine substitutants

In the format provided by the authors and unedited

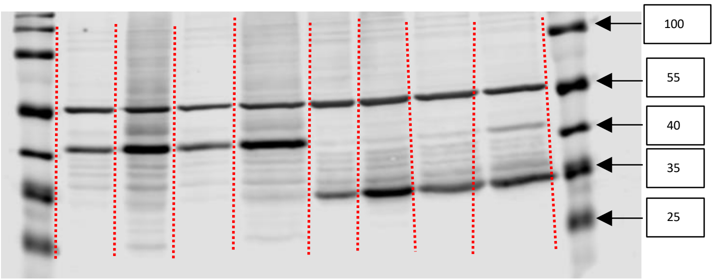


Extended Data 1e: Staining of Tubulin + V5

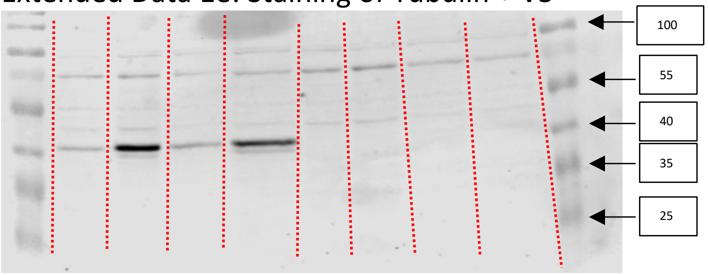


Extended Data 1e: Staining of tGFP

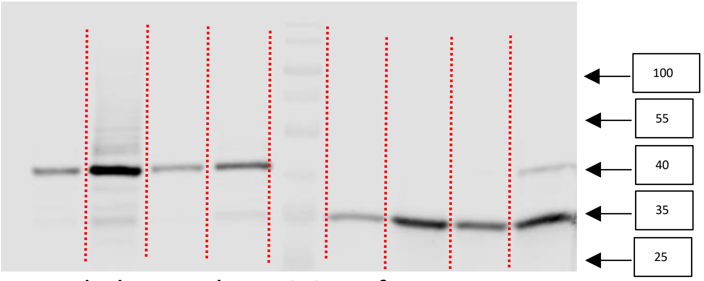


Extended Data 1b: Staining of V5

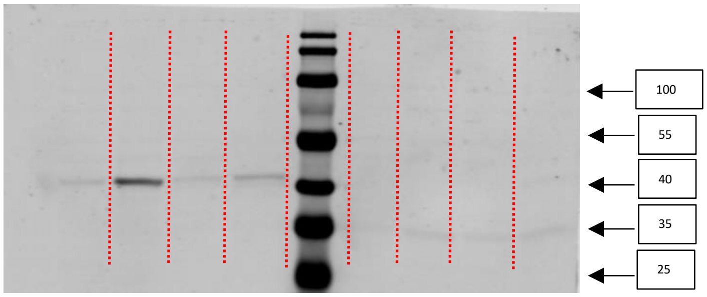


Extended Data 1B: Staining of tGFP

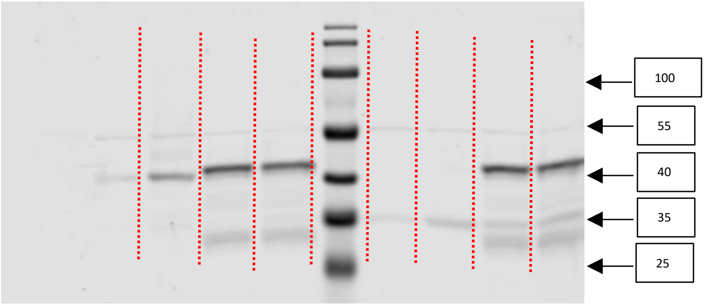


Extended Data 1b: Staining of IDO

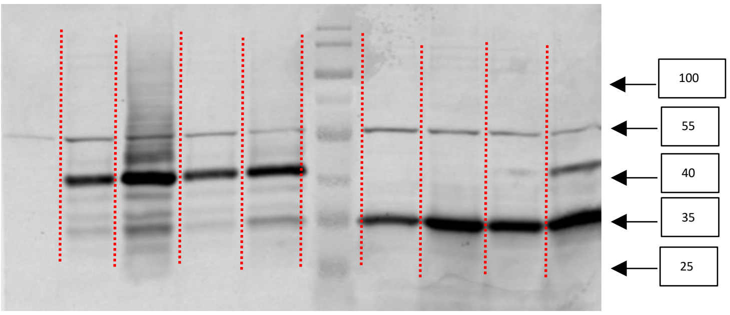

Extended Data 1b: Staining of Tubulin

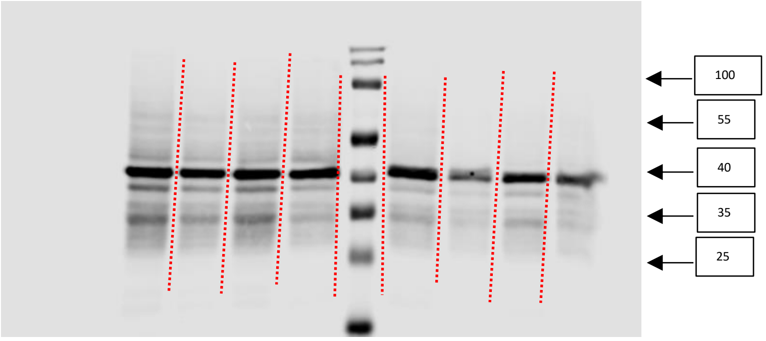

Extended Data 5c: Staining of V5

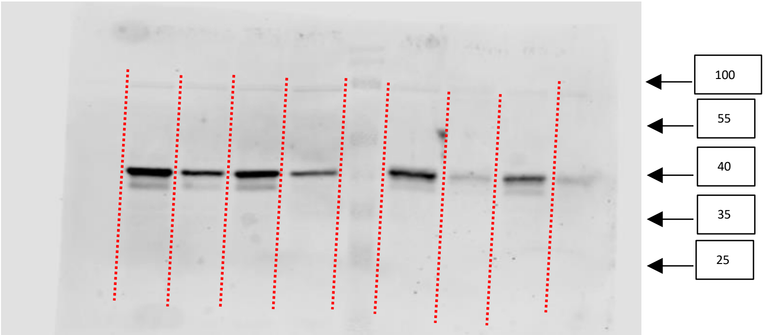

Extended Data 5c: Staining of tGFP

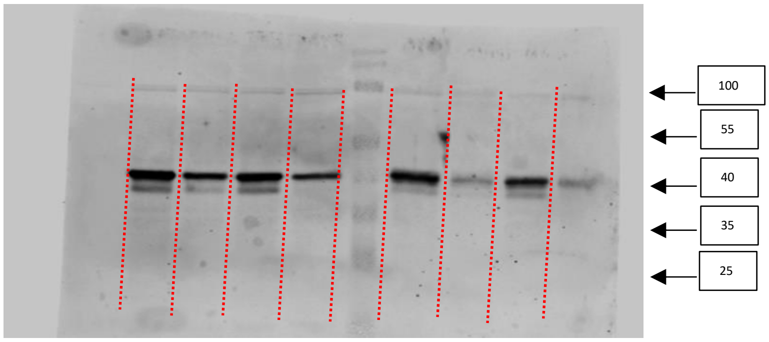

Extended Data 5c: Staining of HSP90

Gating strategy FACS:

Fig 1e:

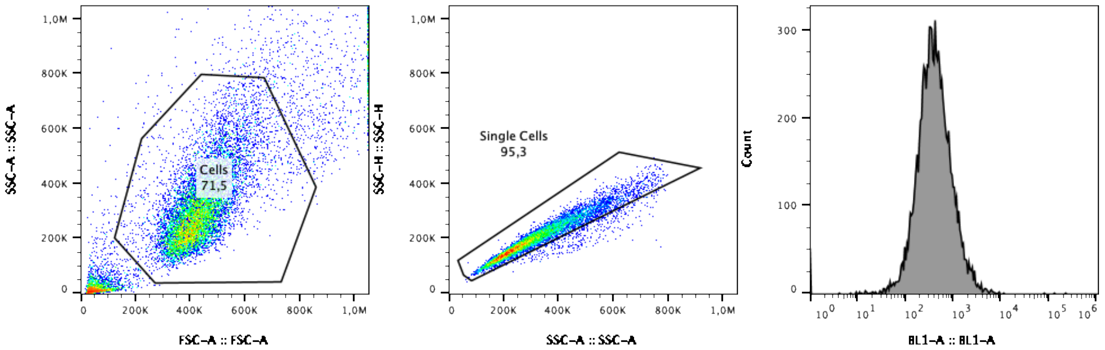


Fig 4b:

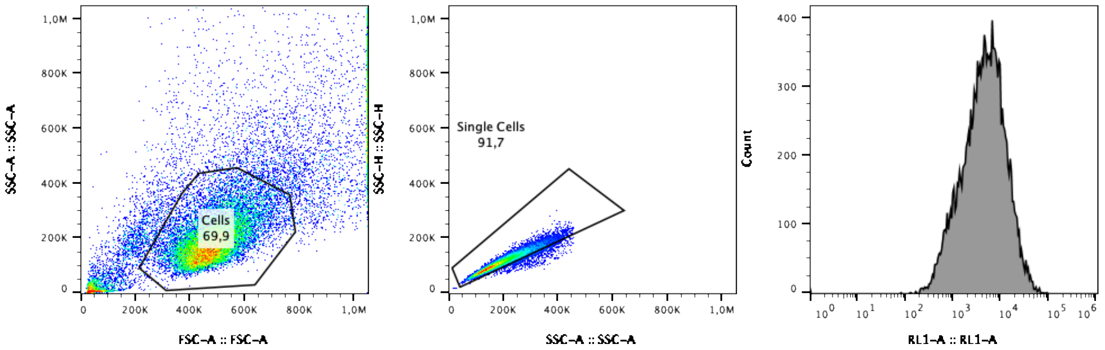

Supplementary Data 3

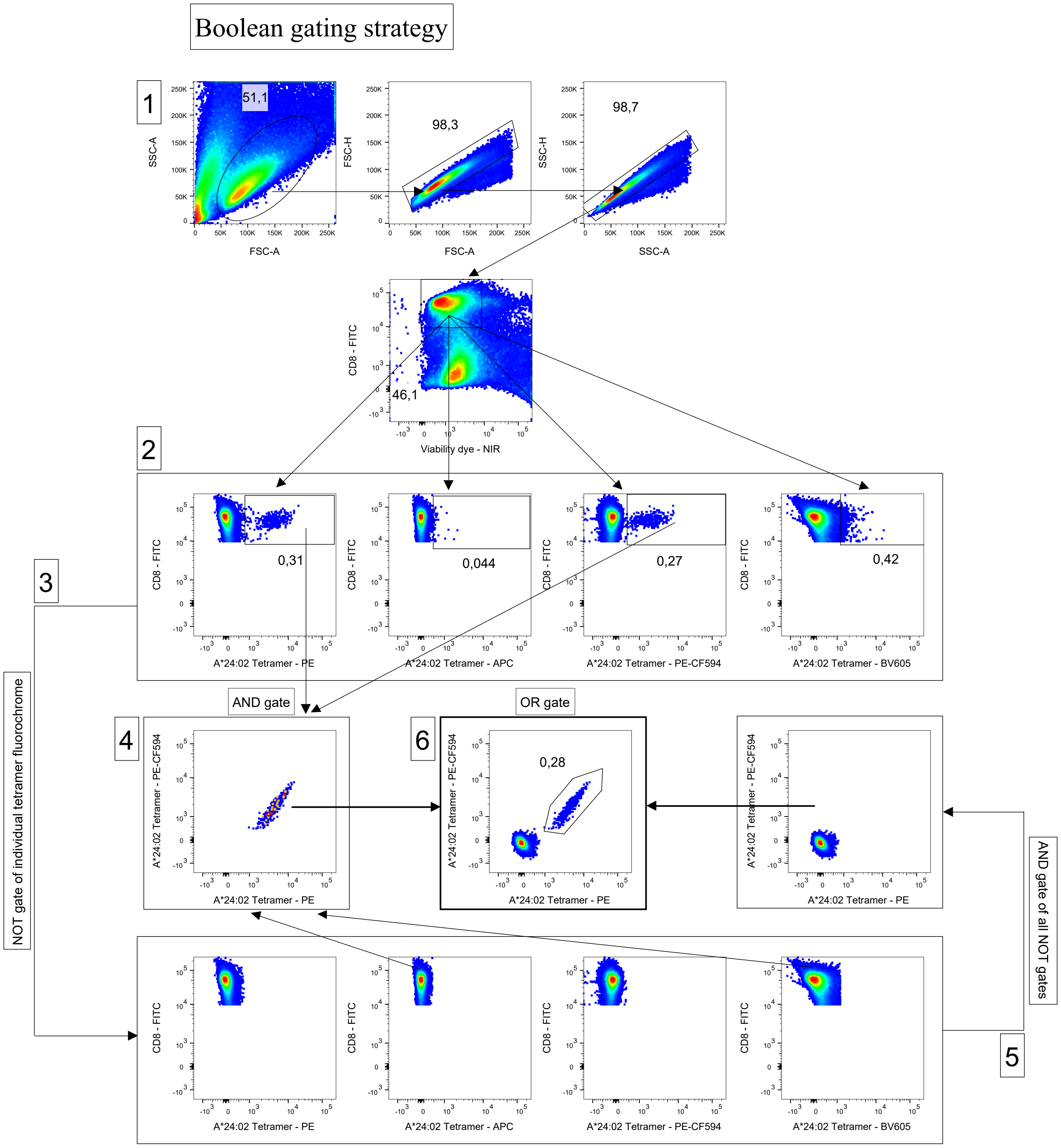


Supplementary Figure 3

Boolean gating analysis of co-cultures with naive CD8+ T cells and autologous monocyte-derived dendritic cells pulsed with substitutant peptides. 1) Lymphocytes are gated and viable CD8+ T cells selected for Boolean gating following doublet exclusion. 2) The pMHC multimer positive population is defined for each individual HLA-A*24:02 multimer fluorochrome among viable CD8+ T cells. 3) NOT gates are made to obtain CD8+/HLA-A*24:02 multimer-fluorochromeneg cells for each multimer-fluorochrome. 4) AND gates are made by combining two HLA-A*24:02 multimer-fluorochromepos populations from step 2 and the NOT gates for remaining multimer fluorochromes from step 3. This step selects for cells that are only positive for two multimer fluorochromes. Cells that are positive for only one, or more than two, multimer fluorochrome(s) are gated out. These AND gates are made for all possible two-color multimer-fluorochrome combinations. 5) An AND gate is made for all multimer-fluorochrome NOT gates made in step 3. 6) An OR gate is made by combining the AND gates from step 4 and 5. The final plot now shows CD8+ T cells that are either negative for all multimer-fluorochromes or positive for the specific combination of two multimer-fluorochromes.
