# Supplementary material for: Tryptophan depletion results in tryptophan-to-phenylalanine substitutants
Source: Nature. 2022 Mar 9;603(7902):721–7. doi: 10.1038/s41586-022-04499-2 (PMC8942854; doi:10.1038/s41586-022-04499-2)
Supplement: Supplementary file 2 — Reporting Summary [file 41586_2022_4499_MOESM2_ESM.pdf]

nature portfolio

Corresponding author(s): Reuven Agami

Last updated by author(s): 24/12/21

Reporting Summary

Nature Portfolio wishes to improve the reproducibility of the work that we publish. This form provides structure for consistency and transparency in reporting. For further information on Nature Portfolio policies, see our Editorial Policies and the Editorial Policy Checklist.

Statistics

For all statistical analyses, confirm that the following items are present in the figure legend, table legend, main text, or Methods section.

n/a	Confirmed	
☐	☒	The exact sample size (n) for each experimental group/condition, given as a discrete number and unit of measurement
☐	☒	A statement on whether measurements were taken from distinct samples or whether the same sample was measured repeatedly
☐	☒	The statistical test(s) used AND whether they are one- or two-sided<br>*Only common tests should be described solely by name; describe more complex techniques in the Methods section.*
☒	☐	A description of all covariates tested
☒	☐	A description of any assumptions or corrections, such as tests of normality and adjustment for multiple comparisons
☐	☒	A full description of the statistical parameters including central tendency (e.g. means) or other basic estimates (e.g. regression coefficient) AND variation (e.g. standard deviation) or associated estimates of uncertainty (e.g. confidence intervals)
☐	☒	For null hypothesis testing, the test statistic (e.g. F, t, r) with confidence intervals, effect sizes, degrees of freedom and P value noted<br>*Give P values as exact values whenever suitable.*
☒	☐	For Bayesian analysis, information on the choice of priors and Markov chain Monte Carlo settings
☒	☐	For hierarchical and complex designs, identification of the appropriate level for tests and full reporting of outcomes
☐	☒	Estimates of effect sizes (e.g. Cohen's d, Pearson's r), indicating how they were calculated

Our web collection on statistics for biologists contains articles on many of the points above.

Software and code

Policy information about availability of computer code

Data collection	Flow cytometry data was acquired using Attune Nxt software version 4.2.<br>All other data collection was implemented manually, without use of external softwares. The sources are described in the manuscript
Data analysis	MaxQuant v1.6.0.16 was used for Proteomics analysis. Proteomics file formal conversion was undertaken using MSCONVERT from proteowizard package (release 3.0.10827). PEPQUERY (v1.6.0) was used for large scale proteomics scanning of peptides. All other analysis was undertaken using custom codes (https://github.com/apataskar/substitutants_manuscript). Code availability statement has been added to the manuscript<br>For prediction of peptide binding strength NetMHCpan 4.0 software was used.<br>FlowJo V10 software was used for analysis of FACS data.

For manuscripts utilizing custom algorithms or software that are central to the research but not yet described in published literature, software must be made available to editors and reviewers. We strongly encourage code deposition in a community repository (e.g. GitHub). See the Nature Portfolio guidelines for submitting code & software for further information.

Data

Policy information about availability of data

All manuscripts must include a data availability statement. This statement should provide the following information, where applicable:

- Accession codes, unique identifiers, or web links for publicly available datasets
- A description of any restrictions on data availability
- For clinical datasets or third party data, please ensure that the statement adheres to our policy

The mass spectrometry proteomics data have been deposited to the ProteomeXchange Consortium via PRIDE partner repository with the dataset identifier

PXD022707. The following publicly available datasets were used for this study; Proteomics Data Commons –PDC Study Identifier (PDC000234, PDC000270,PDC000198, PDC000221, PDC000173, PDC000204, PDC000110, PDC000116, PDC000153, PDC000303) and PRIDE datasets (PXD020079, PXD020224, PXD022707). The UNIPROT Database is sourced from UNIPROT.org with following identifier UP000005640.

Field-specific reporting

Please select the one below that is the best fit for your research. If you are not sure, read the appropriate sections before making your selection.

☒ Life sciences ☐ Behavioural & social sciences ☐ Ecological, evolutionary & environmental sciences

For a reference copy of the document with all sections, see nature.com/documents/nr-reporting-summary-flat.pdf

Life sciences study design

All studies must disclose on these points even when the disclosure is negative.

Sample size	No sample size calculation was performed.<br>For proteomics datasets; atleast two biological replicates were used based on previoud reports in the field.<br>For immunopeptidomics datasets; atleast two biological replicates were used based on previoud reports in the field.<br>The analysis of patient data employed usage of datasets published in peer-reviewed journals in entirety.
Data exclusions	No datasets were excluded
Replication	All data was analyzed in at least n=2 biological replicates, and the number of replicates are mentioned clearly in the manuscript. No replicates were excluded from the analysis or from reporting in the manuscript.<br>For all the figures related to flow cytometry experiments, 3 biological replicates were used (n=3). The individual value of each replicates are displayed by a dot on each figures as well as the standard deviation (SD). For the Western Blot figures, experiments were performed at least 2 independent times, and for clarity, only one of them is showned in the paper.
Randomization	For all high throughput data analysis- randomization for allocation of samples into experimental groups is not relevant, as the design of the study requires explorative analysis for identifying discriminative features between already established experimental groups.
Blinding	For all high throughput data analysis- blinding to investigators is not relevant to this study, as the design of the study requires explorative analysis for identifying discriminative features.

Reporting for specific materials, systems and methods

We require information from authors about some types of materials, experimental systems and methods used in many studies. Here, indicate whether each material, system or method listed is relevant to your study. If you are not sure if a list item applies to your research, read the appropriate section before selecting a response.

Materials & experimental systems

n/a	Involved in the study	
☐	☒	Antibodies
☐	☒	Eukaryotic cell lines
☒	☐	Palaeontology and archaeology
☐	☒	Animals and other organisms
☐	☒	Human research participants
☒	☐	Clinical data
☒	☐	Dual use research of concern

Methods

n/a	Involved in the study	
☒	☐	ChIP-seq
☐	☒	Flow cytometry
☒	☐	MRI-based neuroimaging

Antibodies

Antibodies used	IDO (D5J4E™) Cell Signaling #86630 (RRID: AB_2636818) 1:1000 ; V5 Tag Monoclonal Antibody Thermo-Fisher Scientific #R960-25 (RRID: AB_2556564) 1:5000 ; Anti-α Tubulin Antibody (YL1/2) Santa Cruz #sc-53029 (RRID: AB_793541) 1:20 000 ; TurboGFP Polyclonal Antibody ThermoFisher #PA5-22688 (RRID: AB_2540616) 1:1000<br>APC anti-mouse H-2Kb bound to SIINFEKL Antibody (Clone 25-D1.16) Biolegend #141606 (RRID: AB_11219595) 1:200; CD8b Antibody, anti-mouse, REAfinity™ (Clone REA793) Miltenyi Biotec #130-111-638 (RRID: AB_2659542) 1:100; IFN-γ Antibody, antimouse<br>(Clone AN.18.17.24) Miltenyi Biotec #130-109-723 (discontinued) and #130-120-805 (RRID: AB_2784369) 1:100; TNF-α Antibody, anti-mouse (Clone MP6-XT22) #130-109-719 (discontinued) and #130-102-386 (RRID: AB_2661141) 1:100<br>Secondary antibodies:<br>IRDye® 680RD Donkey anti-Mouse IgG Secondary Antibody Li-COR #926-68072 1:10 000; IRDye® 800CW Goat anti-Rat IgG Secondary Antibody Li-COR #926-32219 1:10 000; IRDye® 800CW Goat anti-Rabbit IgG Secondary Antibody Li-COR #926-32211 1:10 000
Validation	All antibodies used in the study were validated by the manufacturer. Those data are available at the manufacturer website listed below:<br>-IDO (D5J4E™): https://www.cellsignal.com/products/primary-antibodies/ido-d5j4e-rabbit-mab/86630<br>3<br>nature portfolio | reporting summary March 2021<br>-V5 Tag Monoclonal Antibody: https://www.thermofisher.com/antibody/product/V5-Tag-Antibody-Monoclonal/R960-25<br>-Anti-α Tubulin Antibody (YL1/2): https://www.scbt.com/p/alpha-tubulin-antibody-yl1-2<br>-TurboGFP Polyclonal Antibody: https://www.thermofisher.com/antibody/product/TurboGFP-Antibody-Polyclonal/PA5-22688<br>-APC anti-mouse H-2Kb bound to SIINFEKL Antibody: https://www.biolegend.com/en-us/products/apc-anti-mouse-h-2kb-bound-tosiinfekl-<br>antibody-7882<br>-CD8b Antibody, anti-mouse, REAfinity™: https://www.miltenyibiotec.com/NL-en/products/cd8b-antibody-anti-mouse-reafinityrea793.<br>html?countryRedirected=1#gref<br>-IFN-γ Antibody, anti-mouse: https://www.miltenyibiotec.com/NL-en/products/ifn-g-antibody-anti-mouse-an-18-17-24.html#gref<br>-TNF-α Antibody, anti-mouse: https://www.miltenyibiotec.com/NL-en/products/tnf-a-antibody-anti-mouse-mp6-xt22.html#gref<br>-IRDye® 680RD Donkey anti-Mouse IgG Secondary Antibody: https://www.licor.com/bio/reagents/irdye-680rd-donkey-anti-mouseigg-<br>secondary-antibody<br>-IRDye® 800CW Goat anti-Rat IgG Secondary Antibody: https://www.licor.com/bio/reagents/irdye-800cw-goat-anti-rat-iggsecondary-antibody<br>-IRDye® 800CW Goat anti-Rabbit IgG Secondary Antibody: https://www.licor.com/bio/reagents/irdye-800cw-goat-anti-rabbit-iggsecondary-<br>antibody

Eukaryotic cell lines

Policy information about cell lines

Cell line source(s)	MD55A3 cells are given by Prof. Jennifer Wargo and already used in a previous publication (PMID: 33328638). 293T (CRL-3216), HCT-116 (CCL-247), MDA-MB-231 (HTB-26), MCF10A (CRL-10317), MCF7 (HTB-22) and RPE-1 (CRL-4000) are coming from in-house stocks and originally from ATCC.<br>OT-1 cells were isolated freshly from mice. Details of the procedure are presented in the material and method section.
Authentication	Cell lines obtained directly from ATCC. No authentification was performed
Mycoplasma contamination	All cell lines tested negative for mycoplasma in a PCR-based assay
Commonly misidentified lines<br>(See ICLAC register)	No commonly misidentified cell lines were used

Animals and other organisms

Policy information about studies involving animals; ARRIVE guidelines recommended for reporting animal research

Laboratory animals	OT-I (B6J) mice were originally from The Jackson Laboratory, Bar Harbor, ME. Mice used for experiments were between 3 wk and 12 wk old and of both genders.
Wild animals	No wild animals were used in the study
Field-collected samples	No field collected samples were used in the study
Ethics oversight	All experiments involving animals were performed in accordance with Dutch and European regulations on care and protection of

laboratory animals and have been approved by the local animal experiment committee at Netherlands Cancer Institute, DEC NKI (OZP ID 12051).

Note that full information on the approval of the study protocol must also be provided in the manuscript.

Human research participants

Policy information about studies involving human research participants

Population characteristics	We received buffy-coats from anonymous healthy blood donors from Oslo University Hospital Blood Bank in accordance with the institutional guidelines. In addition, we processed and HLA-typed blood from recruited healthy in-house donors under informed consent. All enlisted donors are healthy persons residing in Norway during the study period. For donors provided by Oslo University Hospital Blood Bank we do not have data available for donor age, gender, ethnicity or disease profile. For recruited healthy in-house donors their name, date of birth, hemoglobin and ferritin levels, and HLA type were recorded. All the information was de-identified and each donor was assigned a unique code that connects information through a list of names stored in a secure location
Recruitment	HLA-A*24:02 positive buffy-coats from the blood bank were used without any further selection criteria for donor age, gender or ethnicity. In-house donors were selected based on their HLA type.
Ethics oversight	PBMCs were isolated from the blood of healthy donors donated to the Norwegian Blood Bank under informed consent. Inhouse<br>donor PBMCs were isolated from the healthy donors under informed consent and HLA-typed.

Note that full information on the approval of the study protocol must also be provided in the manuscript.

Flow Cytometry

Plots

Confirm that:

- [x] The axis labels state the marker and fluorochrome used (e.g. CD4-FITC).
- [x] The axis scales are clearly visible. Include numbers along axes only for bottom left plot of group (a 'group' is an analysis of identical markers).
- [x] All plots are contour plots with outliers or pseudocolor plots.
- [x] A numerical value for number of cells or percentage (with statistics) is provided.

Methodology

Sample preparation	Cells were harvested by trypsinization, and centrifugation after which the cells were immediately analyzed on FACS for GFP positivity using Attune Nxt.<br>For SIINFEKL experiments, cells were harvested using PBS-EDTA and stained using APC-conjugated anti-H2-Kb-bound SIINFEKL antibodies.<br>For T-cell (OT-1) activation, T-cells were harvested 12 hours after co-culture with MD55A3 cells by centrifugation in V-shape 96 wells plate. Then cells were stained with anti-mouse CD8-VioBlue antibodies and Live/Dead Fixable near-IR dead cell stain kit. Subsequently, the cells were fixed and permeabilized using the eBioscience™ Foxp3 Transcription Factor Staining Buffer Set. Next, the cells were stained with APC-conjugated anti-mouse IFNγ and PE-conjugated anti-mouse TNFα antibodies. Cells were then washed and analyzed on a BD LSR Fortessa (BD Biosciences).
Instrument	Attune Nxt, BD LSR Fortessa, FACSAria Fusion
Software	Attune Nxt Software or FACSDiva (BD LSR Fortessa) were used for acquisition, FlowJo 10 software was used for data analysis
Cell population abundance	No sorting was applied for downstream use, except for the isolation of the top 7.5% presenting cells of HT29 SIINwEKL cells.
Gating strategy	For all the Flow cytometry related experiments, cells were selected based on FCS and SSC gates. then, single cells were gated out using SSC-A vs SSC-H dot plots.<br>For OT-1 cell activity, after gating the single cells, live cells were selected based on the Live/Dead staining. From this population, CD8+ cells were finally selected. In consecutive experiments, gates for IFN and TNF were drawn based on clear separation between negative and positive populations.

- [x] Tick this box to confirm that a figure exemplifying the gating strategy is provided in the Supplementary Information.
